# Roxadustat for CKD-related anemia in patients undergoing peritoneal dialysis: a systematic review and meta-analysis

**DOI:** 10.1186/s12882-025-04723-x

**Published:** 2026-05-25

**Authors:** Omar Elkoumi, Ahmed Elkoumi, Mariam Khaled Elbairy, Mostafa Adel T. Mahmoud, Hamza Irfan

**Affiliations:** 1 Faculty of Medicine, Suez University, Suez, Egypt; 2 Department of Oral and Dental Medicine, Health Affairs Directorate of El Sharqeya, The Ministry of Health and Population, El Sharqeya, Egypt; 3 Faculty of Medicine, Beni Suef University, Beni Suef, Egypt; 4 Department of Medicine, Shaikh Khalifa Bin Zayed Al Nahyan Medical and Dental College, Lahore, Pakistan

**Keywords:** Roxadustat, Peritoneal dialysis, CKD-related anemia, Hemoglobin, Iron metabolism, Hypoxia-inducible factor, Lipid profile

## Abstract

**Background:**

CKD-related anemia remains a major complication in patients receiving peritoneal dialysis (PD), with limited treatment options beyond erythropoiesis-stimulating agents. Roxadustat, an oral hypoxia-inducible factor prolyl hydroxylase inhibitor, has shown promise in correcting CKD-related anemia and modulating iron and metabolic parameters. However, its evidence in PD remains limited.

**Methods:**

We conducted a systematic search of PubMed, Scopus, and Web of Science from inception to July 20, 2025. We included studies reporting the efficacy and safety of roxadustat in PD. Statistical analysis was performed using Review Manager (RevMan 5.4 for Windows) and R Studio.

**Results:**

Eight studies were included in the meta-analysis (*n* = 607). Roxadustat significantly increased hemoglobin at all time points (MD 0.35 g/dL, 95% CI 0.28–0.41; *p* < 0.00001), serum iron (MD 0.95 µmol/L, 95% CI 0.02–1.89; *p* = 0.05), and total iron-binding capacity (MD 6.25 µmol/L, 95% CI 3.95–8.55; *p* < 0.00001), and reduced hepcidin (MD − 12.28 ng/mL, 95% CI − 21.06 to − 3.50; *p* = 0.006). No significant effects were observed for ferritin (*p* = 0.49), transferrin saturation (*p* = 0.45), cholesterol (*p* = 0.07), LDL (*p* = 0.14), HDL (*p* = 0.27), triglycerides (*p* = 0.26), CRP (*p* = 0.75), systolic blood pressure (*p* = 0.10), or diastolic blood pressure (*p* = 0.08). Heterogeneity was low to moderate for most outcomes.

**Conclusion:**

Roxadustat is effective in improving hemoglobin and iron metabolism in patients with PD, while exerting neutral effects on lipids, inflammation, and blood pressure. These findings support its role as a promising therapy for CKD-related anemia in PD.

**Clinical trial number:**

Not applicable.

**Supplementary Information:**

The online version contains supplementary material available at 10.1186/s12882-025-04723-x.

## Introduction

CKD-related anemia is a frequent and clinically significant complication in patients receiving peritoneal dialysis (PD) [1]. It is driven by impaired erythropoietin production, disturbances in iron metabolism, and chronic inflammation [2]. These mechanisms contribute not only to hematologic insufficiency but also to reduced quality of life, higher cardiovascular risk, and increased mortality [3].

Erythropoiesis-stimulating agents (ESAs), including recombinant human erythropoietin (rHuEPO), remain the standard treatment for CKD-related anemia in patients with PD [4]. While effective in increasing hemoglobin levels, their use is often complicated by hyporesponsiveness due to inflammation and functional iron deficiency [5]. High ESA doses have been linked to elevated risks of cardiovascular and cerebrovascular events, raising concerns about long-term safety. These risks are hypothesized to stem from ESAs promoting vasoconstriction (via mechanisms like increased calcium influx in smooth muscle cells), causing an antinatriuretic effect, leading to induced hypertension, and potentially increasing the risk of thrombosis through direct effects on the endothelium [6, 7]. Moreover, the need for parenteral administration imposes an additional burden on patients treated at home with PD. These limitations highlight the unmet need for alternative therapies that are both effective and convenient.

Roxadustat, an oral hypoxia-inducible factor prolyl hydroxylase inhibitor (HIF-PHI), has emerged as a novel therapeutic approach. By stabilizing hypoxia-inducible factor, roxadustat stimulates endogenous erythropoietin production, promotes intestinal iron absorption, enhances iron mobilization, and reduces hepcidin levels [8, 9]. These combined actions may be particularly advantageous in patients with PD, who frequently experience inflammation-driven disturbances in iron metabolism. In addition, several studies have suggested that roxadustat may exert beneficial effects beyond CKD-related anemia correction, including improvements in lipid profiles and modulation of inflammation, with potential implications for cardiometabolic benefits [10–12].

Although clinical studies have examined roxadustat in patients with PD, the evidence remains scattered and inconsistent. Studies differ in their evaluation of hematologic efficacy, iron metabolism, lipid modulation, and cardiovascular outcomes, leaving uncertainty about its overall therapeutic role in this population. To address this gap, we conducted a systematic review and meta-analysis to evaluate the efficacy and safety of roxadustat in patients with PD, focusing on hematologic, iron-related, metabolic, and cardiovascular outcomes.

## Methods

This systematic review and meta-analysis was designed and reported in alignment with the Cochrane Handbook for Systematic Reviews of Interventions and the PRISMA (Preferred Reporting Items for Systematic Reviews and Meta-Analyses) statement [13, 14].

### Literature search

We systematically searched PubMed, Scopus, and Web of Science (WOS) from inception to July 20, 2025, using a combination of terms for roxadustat, peritoneal dialysis, and CKD-related anemia. Detailed search strategies for each database are provided in Supplementary Table 1. No restrictions were applied to study design, participant characteristics, or publication date.

### Study selection

Studies were eligible if they met the following criteria: (1) published in English; (2) included patients with CKD-related anemia undergoing peritoneal dialysis; (3) evaluated roxadustat; and (4) compared outcomes against erythropoiesis-stimulating agents (ESAs), erythropoietin, or placebo. Eligible study designs included both clinical trials and observational studies. Outcomes of interest were improvement in hemoglobin levels, CKD-related anemia correction, lipid profile, and blood pressure changes. No restrictions were applied regarding age, sex, study location, or publication date. The following studies were excluded: (1) animal or in vitro studies; (2) reviews, book chapters, theses, editorials, letters, or conference abstracts; (3) publications with abstract-only data; (4) studies lacking a control group; and (5) studies in which patients transitioned from recombinant erythropoietin (rhEPO) to roxadustat without a distinct comparator arm. The included studies differed in prior ESA exposure. Some studies enrolled only ESA-naïve patients, others enrolled only ESA-converted patients, and others enrolled mixed cohorts of naïve and converted patients. Comparator groups reflected these differences, ranging from ESA initiation in naïve populations to continued ESA therapy in converted or mixed cohorts. In cases of overlapping populations, priority was given to the study with the longer duration of CKD-related anemia before roxadustat initiation. If this criterion was similar across studies, the publication with the larger sample size and detailed outcomes of interest was included. All retrieved records were imported into EndNote Reference Library (EndNote X9 Version, Clarivate, Philadelphia, PA, USA) for duplicate removal. Title/abstract and full-text screening were then performed independently by two reviewers using the Rayyan web application, with the blinding feature activated. Any disagreements were resolved through discussion with the first author.

### Data extraction

Two reviewers independently extracted data using a standardized template, and discrepancies were resolved by consensus with the first author. Study-level characteristics were recorded, including study and publication year, study design, country, period of data collection, sample size, study population, duration of follow-up, roxadustat dose, control details, and primary outcomes. Baseline patient data were also extracted, encompassing demographic features (age, sex, and body mass index), comorbid conditions (diabetes mellitus and hypertension), dialysis-related variables (duration of dialysis), blood pressure (systolic and diastolic), hematologic parameters (hemoglobin), iron metabolism markers (ferritin, serum iron, and transferrin saturation), renal function indices (serum creatinine and blood urea nitrogen), nutritional status (albumin), and lipid profile (total cholesterol, triglycerides, LDL, and HDL).

### Quality assessment

Two reviewers independently assessed the quality of included studies. For randomized controlled trials, we applied the Cochrane Risk of Bias 2 (RoB 2) tool, which evaluates five domains: bias arising from the randomization process, deviations from intended interventions, missing outcome data, measurement of the outcome, and selection of the reported result [15]. Non-randomized studies were assessed using the Risk of Bias in Non-randomized Studies of Interventions (ROBINS-I) tool across seven domains: bias due to confounding, participant selection, classification of interventions, deviations from intended interventions, missing data, measurement of outcomes, and selection of reported results [16]. Any disagreements were resolved by discussion with the first author.

### Data analysis

All statistical analysis was performed using Review Manager (RevMan, version 5.4; Cochrane Collaboration, London, UK). A p-value < 0.05 was considered statistically significant. Continuous outcomes were analyzed as mean differences (MDs) with corresponding 95% confidence intervals (CIs). A fixed-effects model was applied when heterogeneity was negligible. Random-effects model was applied for outcomes with substantial heterogeneity using the DerSimonian–Laird method. Heterogeneity across studies was quantified using the I² statistic, with I² values ≥ 50% or *p* < 0.10 interpreted as evidence of significant variability. Publication bias was evaluated using Doi plots and Egger’s regression test, implemented in R Studio (R Foundation for Statistical Computing, Vienna, Austria).

## Results

### Search results

**Fig. 1:**
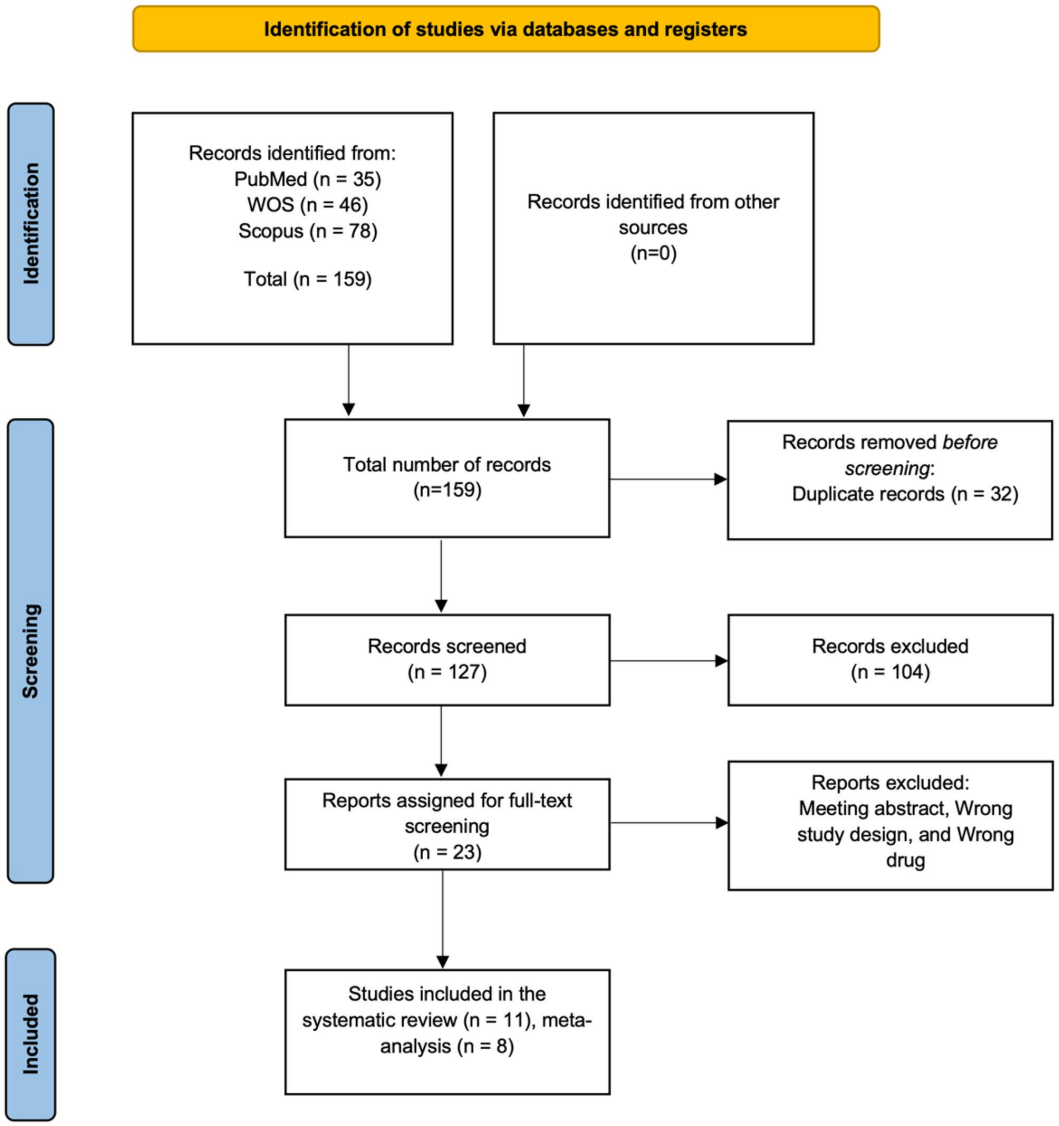
PRISMA flow chart

We identified 159 records through the systematic literature search. After removal of duplicates, 122 articles remained and were screened by title and abstract. Of these, 23 full texts were retrieved for detailed evaluation. Ultimately, 11 studies met the eligibility criteria for inclusion in the systematic review, of which 8 provided sufficient data for quantitative synthesis. Studies were excluded from the meta-analysis if they contained overlapping populations, lacked relevant or adequate outcome data, or reported results in a format unsuitable for pooling. The study selection process is illustrated in Fig. 1.

### Study characteristics

A total of 11 studies published between 2020 and 2025 were included in this systematic review [12, 17–26]; eight provided sufficient data for inclusion in the meta-analysis [12, 18–23, 26]. The included studies comprised both randomized controlled trials (RCTs) and observational designs. Specifically, three were RCTs [12, 25, 26], and the remaining eight were retrospective cohort studies [17–24]. Sample sizes varied considerably, ranging from small single-center cohorts to large multicenter trials. The mean age of participants across studies ranged from approximately 40 to 66 years. The proportion of male patients varied between 31.9% and 76.9%. Body mass index (BMI), where reported, was generally in the normal-to-overweight range (20.7–24.8 kg/m²). Several studies did not provide BMI data.

Comorbid conditions were highly prevalent. Diabetes mellitus was reported in 10%–40% of participants, with some studies identifying diabetic nephropathy as the primary cause of end-stage kidney disease. Hypertension was frequent, with reported prevalence from 33% to over 90%; in some studies, hypertensive nephropathy was specified as the underlying etiology.

Dialysis duration differed markedly, from a median of approximately 10 months to more than 80 months. Baseline systolic blood pressure ranged from 133 to 148 mmHg, and diastolic blood pressure ranged from 81 to 92 mmHg; several studies reported mean arterial pressure instead.

Baseline hemoglobin concentrations were broadly similar across studies, with means between 8.1 and 11.0 g/dL. Iron indices were reported inconsistently, but available data showed mean serum iron concentrations between 10 and 15 µg/dL.

Overall, although study designs varied, the populations were broadly comparable in terms of demographic and clinical characteristics, allowing for meaningful synthesis. A detailed summary of the included studies is presented in Table 1, while Supplementary Table 2 provides the baseline demographic and clinical characteristics of enrolled patients.

**Table 1:** Descriptive summary of included studies

Study and publication year	Study design	Study Setting		Population	Patient number (*n*)		Follow-up duration	Roxadustat dose	Control	Primary outcomes
		Country	Date of collected data		Roxadustat	Control				
Akizawa 2020	Phase 3, randomized, open-label, multicenter trial	Japan	June 2016-August 2017	CKD patients on PD with anemia (ESA-naïve or ESA-converted)	56 in 2 subgroups	No control group	24 weeks	Roxadustat (oral, 50–100 mg TIW based on prior ESA use)	NA, in this study ESA-converted group served as within-study control)	Maintenance rate of Hb 10–12 g/dL (weeks 18–24)
Bao 2022	Retrospective cohort study	China	January 2020-December 2021	CKD patients on PD with anemia, switched from rhEPO due to COVID-19	29	No control group	Median 21.1 months (IQR: 20.6–21.7)	Roxadustat (oral, 100–120 mg TIW based on weight)	No control	Hb change from baseline; medication compliance
Cheng 2023	Retrospective cohort study	China	June 2019 – April 2020 follow-up until March 2022)	18–80 years old, PD patients with renal anemia (Hb < 110 g/L)	60	60	24 months	Roxadustat (oral, 100–120 mg TIW based on weight)	rHuEPO (subcutaneous, dose adjusted per guidelines and other factors)	Blood pressure changes and cardiovascular parameters
Hirai 2021	Retrospective cohort study	Japan	2019–2020	> 20 years PD patients with anemia (CKD stage G5D)	16	23	24 weeks	Roxadustat (oral, TIW at bedtime; mean dose: 189 mg/week at 24 weeks)	ESA (subcutaneous, once/month; darbepoetin alfa or epoetin beta pegol)	Change in hemoglobin and iron profile
Hou 2022	Randomized controlled trial	China	September 2019 – June 2020	PD patients with anemia (Hb < 12 g/dL)	86	43	24 weeks	Roxadustat (oral, 100–120 mg based on weight)	ESA (subcutaneous, continuing prerandomization doses)	Mean Hb at week 24, Hb change from baseline,
Liu 2024	Retrospective cohort study	China	January 2019 – May 2023	18–75 years old PD patients for more than 12 weeks with erythropoietin hyporesponsiveness	61	20	16 weeks	Roxadustat (oral, 100–120 mg TIW based on weight)	ESA (dose/frequency increased, max 2× stable dose)	Change in hemoglobin (Hb) levels at week 16
Liu 2025	Retrospective cohort study	China	December 2022 – January 2023	18–75 years old PD patients for more than 3 months with renal anemia	34	120	9 months	Roxadustat (oral, 100–120 mg TIW based on weight)	ESA (subcutaneous, dose adjusted per guidelines)	Change in hemoglobin (Hb) levels, medication adherence, and COVID-19 outcomes
Wu 2022	Retrospective cohort study	China	November 2019 – October 2020	18–75 years old ESRD patients new to PD with renal anemia	28	32	40 weeks	Roxadustat with Initial dose: 100–120 mg TIW (weight-based); adjusted to maintain Hb 10–12 g/dL	rhuEPO: 50–150 IU/kg/week subcutaneously; target Hb 10–12 g/dL	Change in Hb levels and residual renal function
Xu 2025	Prospective, multicenter, randomized controlled trial	China	June 2020 – November 2021	> 18 years old PD patients for a minimum of 3 months with anemia (stable on EPO for ≥ 4 weeks)	50	50	48 weeks	Oral roxadustat (70 mg or 100 mg TIW, weight-based); target Hb 10–12 g/dL	Subcutaneous EPO (any brand).	Changes in blood lipids and hemoglobin
Zhang 2023	Prospective cohort study	China	June 2021 – April 2022	18–75 years old PD patients for > 6 weeks with anemia	106	53	24 weeks	Oral roxadustat (70–120 mg TIW, based on weight.	ESA (epoetin alfa, 75–100 IU/kg/week subcutaneously)	Changes in iron biomarkers (hepcidin, TSAT, ferritin) and Hb levels
Zhu 2021	Retrospective cohort study	China	-	CKD patients on PD with renal anemia	31 (29 completed follow-up)	No control group	Mean 6.2 ± 3.2 months (range: 3–6 months post-roxadustat initiation)	Roxadustat (oral, 100–120 mg TIW based on weight; mean dose: 86.2 ± 21.3 mg)	No control. It is Within-group comparison.	Hb compliance rate (Hb ≥ 110 g/L)

Abbreviations: CKD: chronic kidney disease; PD: peritoneal dialysis; Hb: hemoglobin; ESA: erythropoiesis-stimulating agent; rhEPO/rHuEPO: recombinant human erythropoietin; ESRD: end-stage renal disease; RRF: residual renal function; TSAT: transferrin saturation; IQR: interquartile range; TIW: three times weekly

### Quality assessment

Two RCTs [12, 26] were judged as having some concerns, mainly related to open-label design and absence of prespecified analysis plans. The observational cohort studies [18–23] were rated moderate due to residual confounding and incomplete adjustment. Overall, the certainty of evidence was considered moderate to some concerns Table 2.

**Table 2:** Risk of bias assessment of included studies in the quantitative synthesis

Study	Design	Tool	Confounding / Randomization	Deviations from interventions	Missing data	Measurement of outcomes	Selection of reported results	Overall RoB
Cheng 2023	Retrospective cohort	ROBINS-I	Moderate (confounding & selection bias possible)	Low	Moderate	Low	Moderate	Moderate
Hirai 2021	Retrospective cohort	ROBINS-I	Moderate (unadjusted confounding)	Low	Moderate	Low	Moderate	Moderate
Hou 2022	Randomized controlled trial	RoB 2	Low (robust randomization)	Some concerns (open-label)	Low (minimal loss)	Low	Some concerns	Some concerns (moderate)
Liu 2024	Retrospective cohort	ROBINS-I	Moderate (confounding, limited control)	Low	Moderate	Low	Moderate	Moderate
Liu 2025	Retrospective cohort	ROBINS-I	Moderate (confounding, limited control)	Low	Moderate	Low	Moderate	Moderate
Wu 2022	Retrospective cohort	ROBINS-I	Moderate (residual confounding)	Low	Moderate (attrition not handled)	Low	Moderate	Moderate
Xu 2025	Randomized controlled trial	RoB 2	Some concerns (allocation concealment unclear)	Some concerns (open-label)	Some concerns (dropouts, ITT not explicit)	Low	Some concerns	Some concerns (moderate)
Zhang 2023	Prospective cohort	ROBINS-I	Moderate (allocation not strictly randomized, residual confounding)	Low	Moderate (attrition not fully reported)	Low	Moderate	Moderate

### Change in Hb

**Fig. 2:**
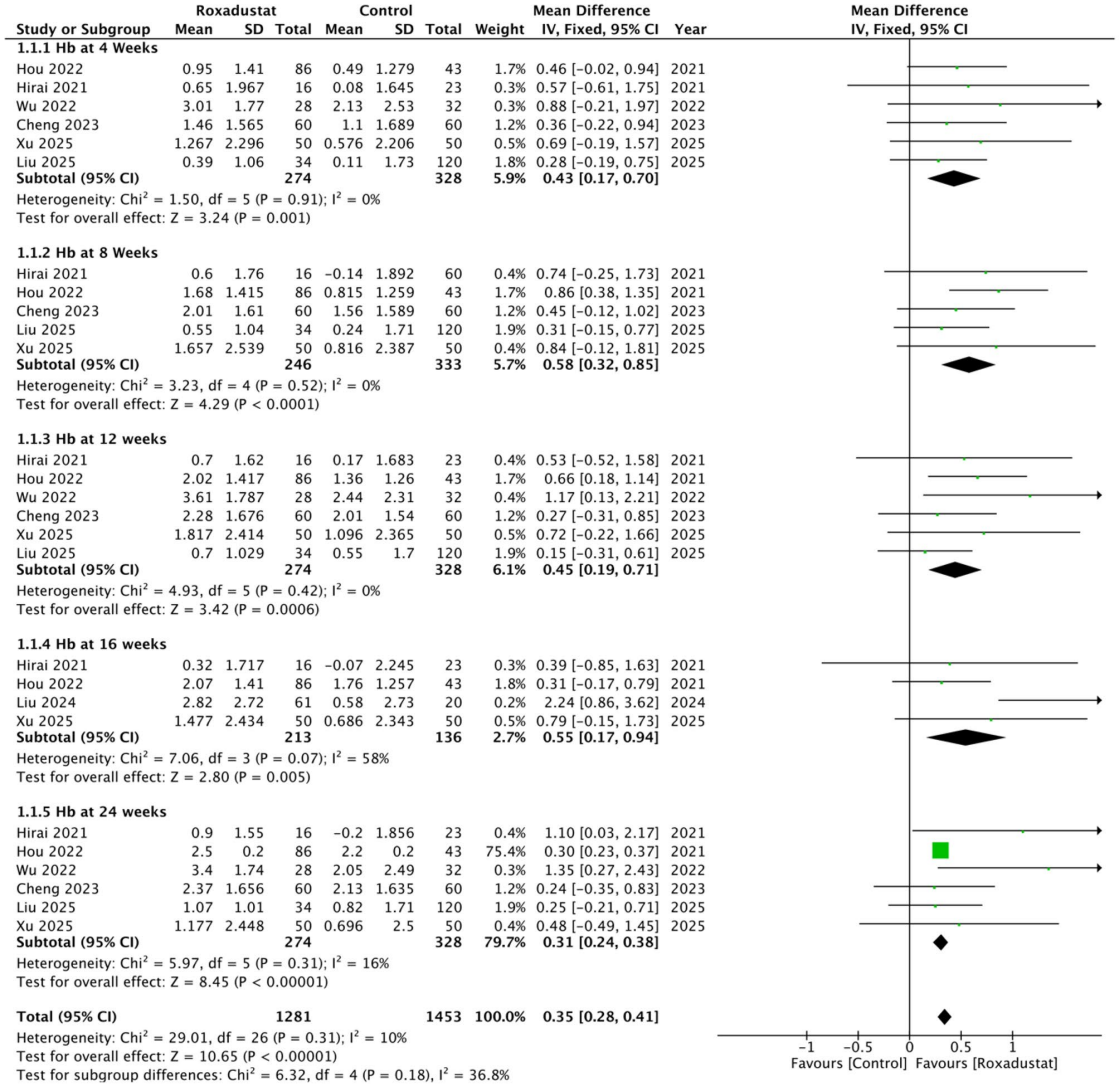
Forest plot of mean differences in hemoglobin (Hb) between roxadustat and control across different follow-up time points

Six studies contributed data on changes in Hb, reported at 27 time points. At 4 weeks, six studies showed a significant difference between roxadustat and control (MD 0.43, 95% CI 0.17 to 0.70; *p* = 0.001; I² = 0%). At 8 weeks, five studies demonstrated a significant effect (MD 0.58, 95% CI 0.32 to 0.85; *p* < 0.0001; I² = 0%). At 12 weeks, six studies demonstrated a significant effect (MD 0.45, 95% CI 0.19 to 0.71; *p* = 0.0006; I² = 0%). At 16 weeks, four studies demonstrated a significant effect (MD 0.55, 95% CI 0.17 to 0.94; *p* = 0.005; I² = 58%). At 24 weeks, six studies demonstrated a significant effect (MD 0.31, 95% CI 0.24 to 0.38; *p* < 0.00001; I² = 16%). Overall, roxadustat was associated with a significant increase in Hb compared with control (MD 0.35, 95% CI 0.28 to 0.41; *p* < 0.00001; I² = 10%), Fig. 2. There was no evidence of subgroup differences. (*p* = 0.18). The funnel plot suggested asymmetry, with Egger’s regression test further confirming evidence of publication bias (t = 4.36, df = 25, *p* = 0.0002), Supplementary Fig. 1.

### Change in Serum Iron

**Fig. 3:**
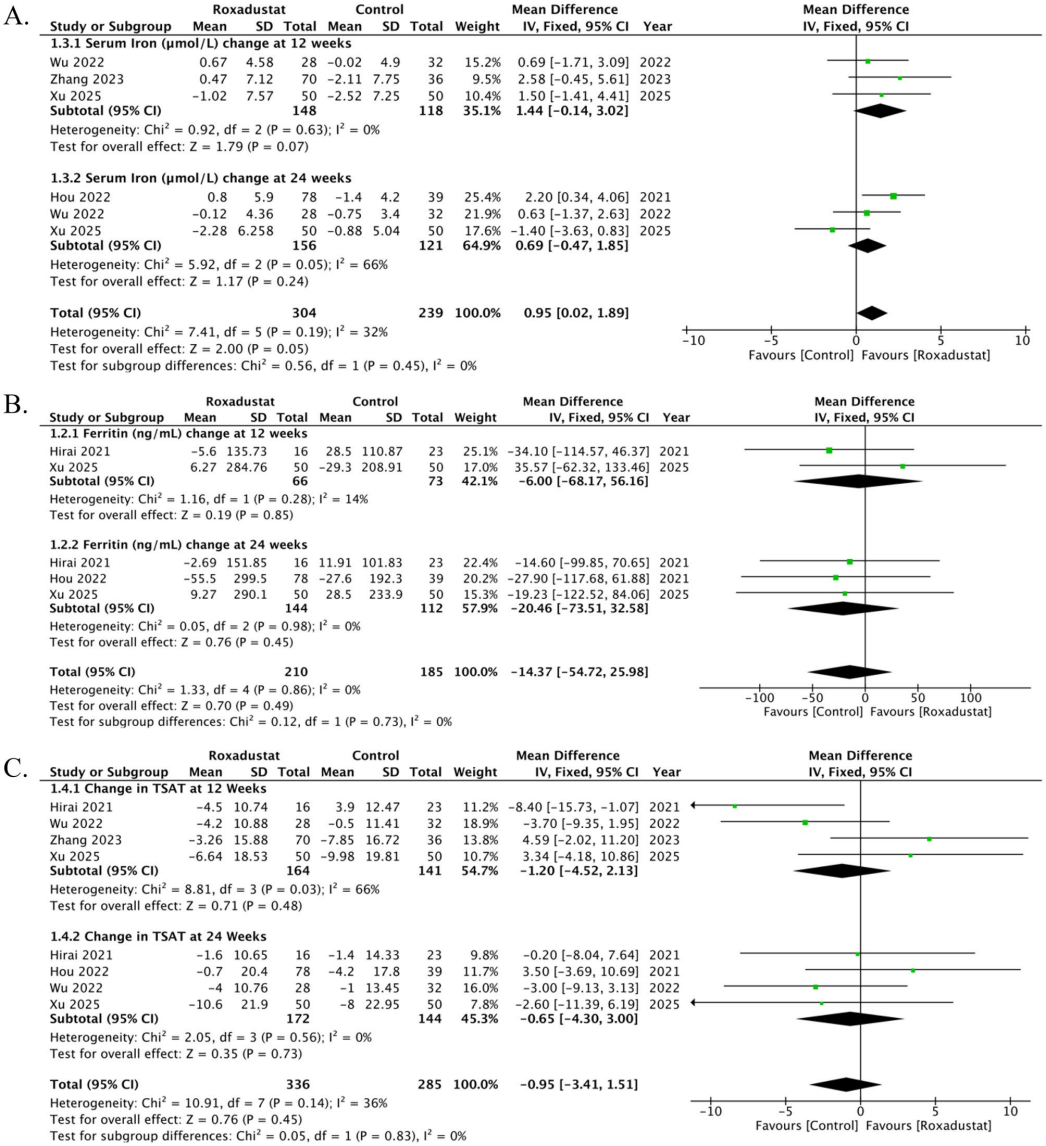

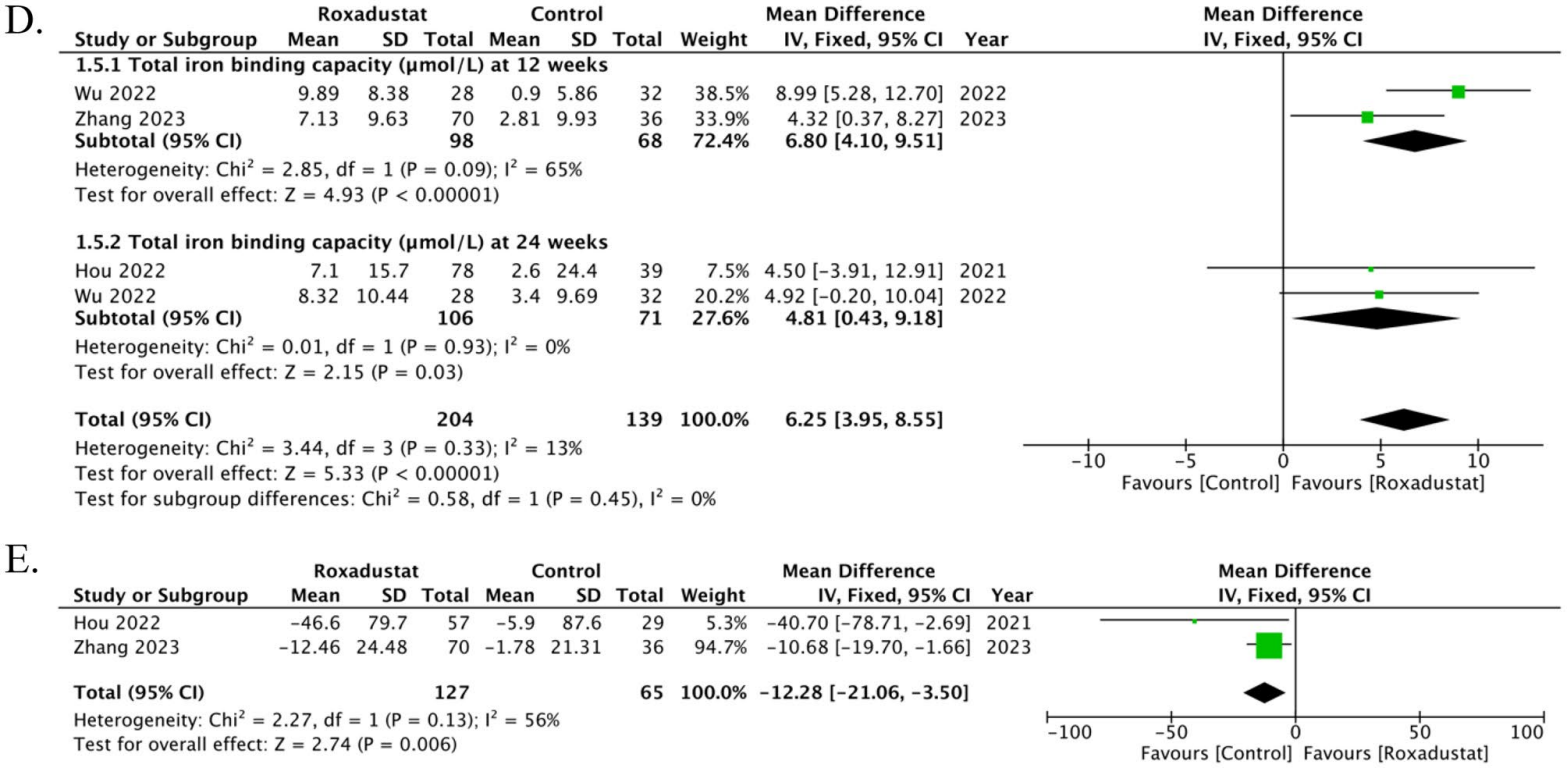
Effects of roxadustat versus control on iron metabolism parameters in peritoneal dialysis patients. (**A**) Change in serum iron: pooled analysis from four studies showed no significant difference at 12 and 24 weeks, although the overall effect indicated a modest increase with roxadustat. (**B**) Change in serum ferritin: Three studies demonstrated no significant difference at 12 or 24 weeks, with the pooled effect suggesting a nonsignificant decrease in ferritin. (**C**) Change in transferrin saturation (TSAT): Five studies reported no significant difference at 12 or 24 weeks, with the overall effect showing a nonsignificant reduction in TSAT. (**D**) Change in total iron-binding capacity (TIBC): Three studies revealed a significant increase at both 12 and 24 weeks, and overall, roxadustat significantly increased TIBC compared with control. (**E**) Change in hepcidin: Two studies showed a significant decrease in hepcidin with roxadustat

Four studies contributed data on changes in serum iron, reported at six time points. At 12 weeks, three studies showed no significant difference between roxadustat and control (MD 1.44, 95% CI − 0.14 to 3.02; *p* = 0.07; I² = 0%). At 24 weeks, three studies also demonstrated no significant effect (MD 0.69, 95% CI − 0.47 to 1.85; *p* = 0.24; I² = 66%). Overall, roxadustat was associated with a significant increase in serum iron compared with control (MD 0.95, 95% CI 0.02 to 1.89; *p* = 0.05; I² = 32%), with no evidence of subgroup differences between (*p* = 0.45), Fig. 3A. The funnel plot appeared symmetrical, suggesting no obvious evidence of publication bias. Egger’s regression test further confirmed the absence of small-study effects (t = 0.22, df = 4, *p* = 0.84), Supplementary Fig. 2.

### Change in Serum Ferritin

Three studies contributed data on changes in serum ferretin, reported at five time points. At 12 weeks, three studies showed no significant difference between roxadustat and control (MD -6.00 µmol/L, 95% CI − 68.17 to 56.15; *p* = 0.85; I² = 14%). At 24 weeks, three studies also demonstrated no significant effect (MD -20.45, 95% CI − 73.51 to 32.58; *p* = 0.45; I² = 0%). Overall, roxadustat was associated with a non-significant change in serum ferritin compared with the control (MD -14.37, 95% CI -54.72 to 25.98; *p* = 0.49; I² = 0%), with no evidence of subgroup differences (*p* = 0.45), Fig. 3B. The funnel plot appeared slightly asymmetrical, suggesting some evidence of publication bias; however, Egger’s regression test confirmed the absence of publication bias (t = 1.14, df = 3, *p* = 0.34), Supplementary Fig. 3.

### Change in TSAT

Five studies contributed data on changes in TSAT, reported at 8 time time points (four at 12 weeks and four at 24 weeks). At 12 weeks, four studies showed no significant difference between roxadustat and control (MD -1.20, 95% CI -4.52 to 2.13; *p* = 0.48; I² = 66%). At 24 weeks, four studies demonstrated no significant effect (MD -0.65, 95% CI -4.30 to 3.00; *p* = 0.73; I² = 0%). Overall, roxadustat was associated with non-significant change in TSAT compared with control (MD -0.95, 95% CI -3.41 to 1.51; *p* = 0.45; I² = 36%), with no evidence of subgroup differences between (*p* = 0.83), Fig. 3C. The funnel plot appeared symmetrical, suggesting no evidence of publication bias. Egger’s regression test confirmed the absence of any publication bias (t = 0.38, df = 6, *p* = 0.72), Supplementary Fig. 4.

### Change in TIBC

Three studies contributed data on changes in TIBC, reported at 4 time points. At 12 weeks, two studies showed a significant difference between roxadustat and control (MD 6.80, 95% CI 4.10 to 9.51; *p* < 0.00001; I² = 65%). At 24 weeks, two studies demonstrated a significant effect (MD 4.81, 95% CI 0.43 to 9.18; *p* = 0.03; I² = 0%). Overall, roxadustat was associated with a significant increase in TIBC compared with control (MD 6.25, 95% CI 3.95 to 8.55; *p* < 0.00001; I² = 13%), with no evidence of subgroup differences between (*p* = 0.45), Fig. 3D. The funnel plot appeared symmetrical, suggesting no evidence of publication bias. Egger’s regression test confirmed the absence of any publication bias (t = -0.66, df = 2, *p* = 0.576), Supplementary Fig. 5.

### Change in Hepcidin

Two studies contributed data on changes in Hepcidin. Overall, roxadustat was associated with a significant decrease in Hepcidin compared with control (MD -12.28, 95% CI -21.06 to -3.50; *p* = 0.006; I² = 56%), Fig. 3E. A DOI plot was constructed to assess publication bias, and the LFK index of − 4.84 indicated the presence of major asymmetry, suggestive of significant publication bias, Supplementary Fig. 6.

### Change in Cholesterol

**Fig. 4:**
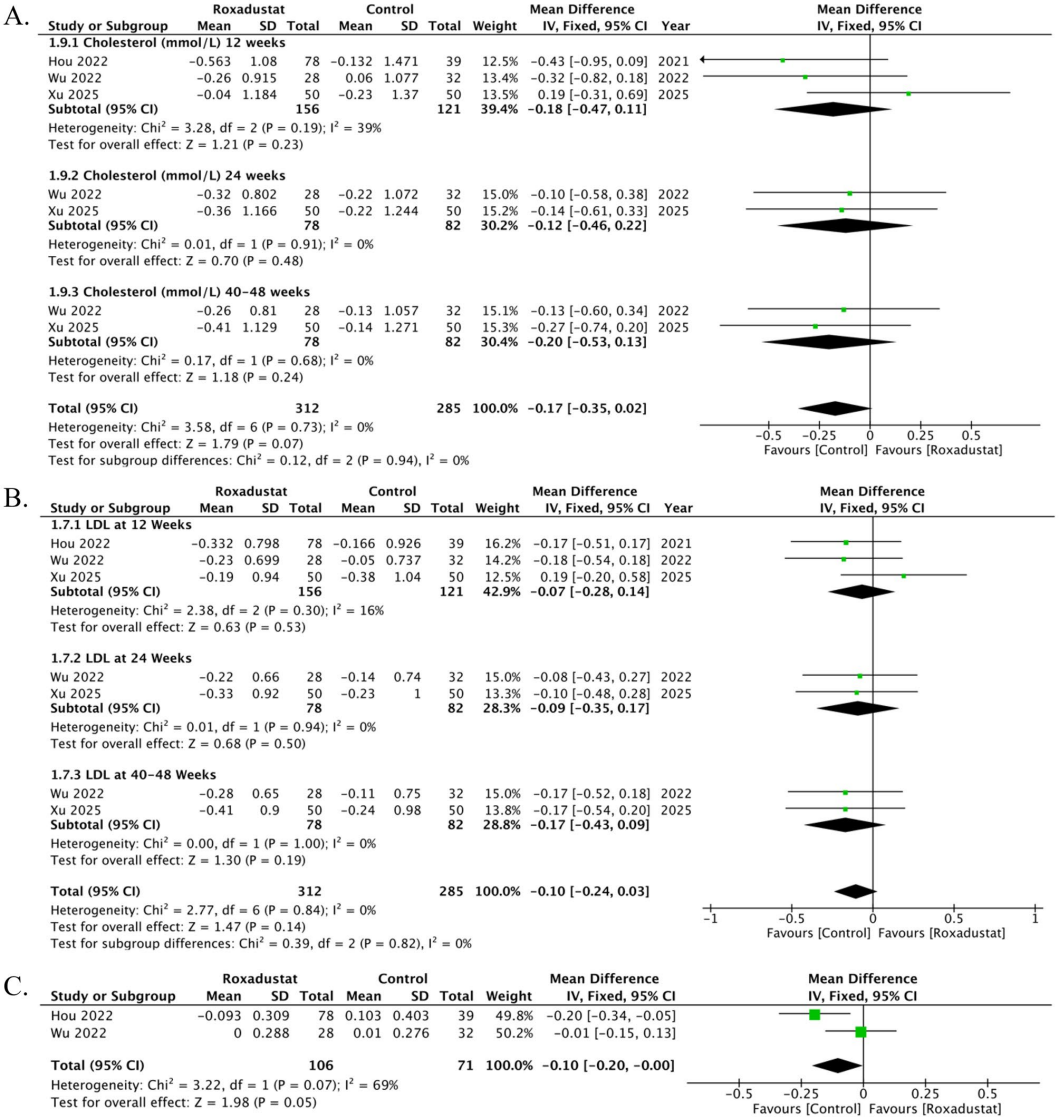

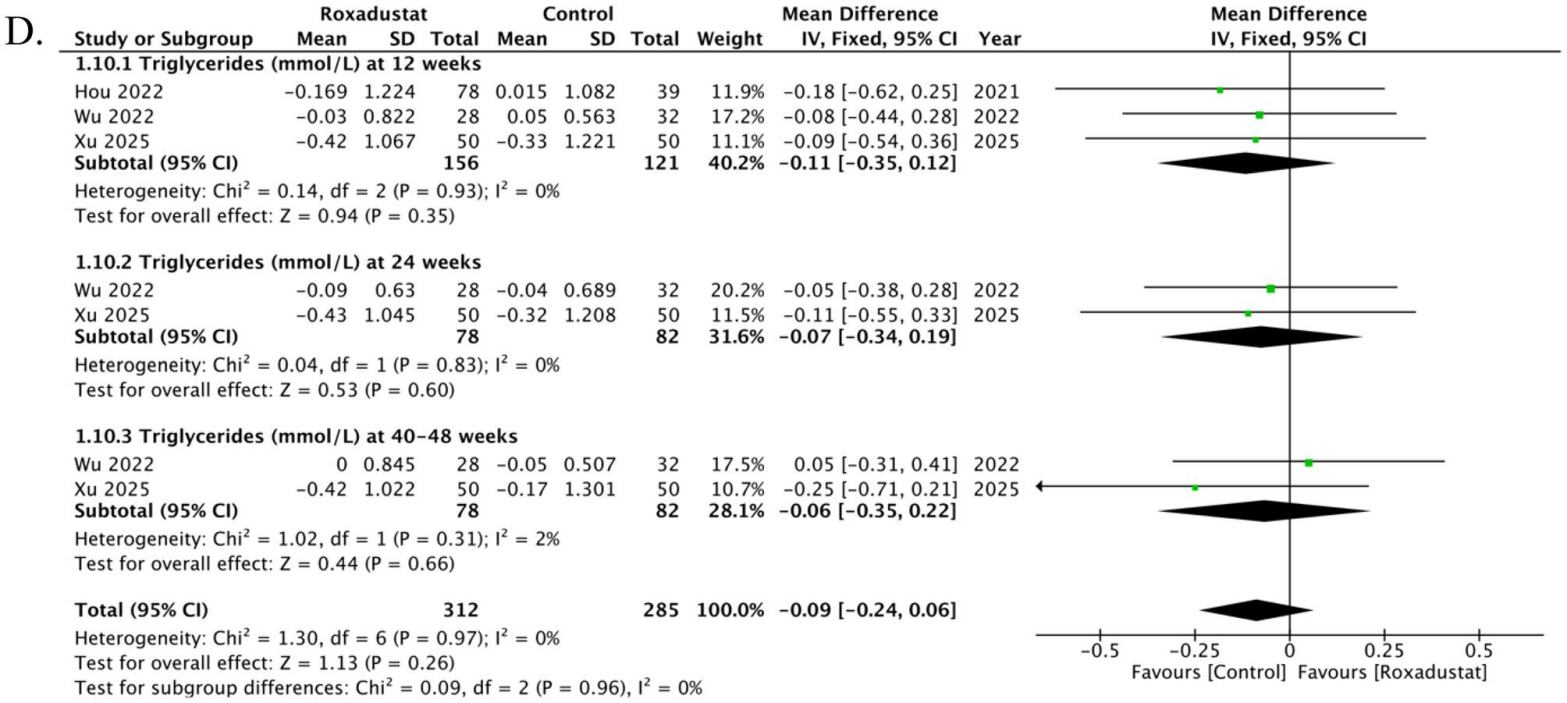
Effects of roxadustat versus control on lipid parameters in peritoneal dialysis patients. (**A**) Change in serum cholesterol: three studies across multiple time points showed a trend toward reduction with roxadustat, but the overall effect was not statistically significant. (**B**) Change in low-density lipoprotein (LDL): Three studies reported a consistent but non-significant reduction in LDL with roxadustat. (**C**) Change in high-density lipoprotein (HDL): Two studies demonstrated no significant difference in HDL, with moderate heterogeneity and some evidence of publication bias. (**D**) Change in triglycerides: Three studies showed a non-significant reduction in triglycerides with roxadustat

Three studies contributed data on changes in serum cholesterol, reported at seven time points. At 12 weeks, three studies showed no significant difference between roxadustat and control (MD -0.18, 95% CI − 0.47 to 0.11; *p* = 0.23; I² = 39%). At 24 weeks, two studies demonstrated no significant effect (MD -0.12, 95% CI − 0.46 to 0.22; *p* = 0.48; I² = 0%). At 40–48 weeks, two studies demonstrated no significant effect (MD -0.20, 95% CI − 0.53 to 0.13; *p* = 0.24; I² = 0%). Overall, roxadustat was associated with a decrease in serum cholesterol compared with control (MD -0.17, 95% CI -0.35 to 0.02; *p* = 0.07; I² = 0%). However, the result was non-significant. There was no evidence of subgroup differences. (*p* = 0.94), Fig. 4A. The funnel plot appeared symmetrical, and Egger’s regression test showed no evidence of small-study effects (t = − 0.53, df = 5, *p* = 0.62), Supplementary Fig. 7.

### Change in LDL

Three studies contributed data on changes in LDL, reported at 7 time points. At 12 weeks, three studies showed a non-significant difference between roxadustat and control (MD -0.07, 95% CI -0.28 to 0.14; *p* = 0.53; I² = 16%). At 24 weeks, two studies demonstrated a non-significant effect (MD -0.09, 95% CI -0.35 to 0.17; *p* = 0.50; I² = 0%). At 40–48 weeks, two studies demonstrated a non-significant effect (MD -0.17, 95% CI -0.43 to 0.09; *p* = 0.19; I² = 0%). Overall, roxadustat was associated with a decrease in LDL compared with control (MD -0.10, 95% CI -0.24 to 0.03; *p* = 0.14; I² = 0%); however, the result was non-significant. There was no evidence of subgroup differences between (*p* = 0.82), Fig. 4B. The funnel plot appeared symmetrical, suggesting no evidence of publication bias. Egger’s regression test confirmed the absence of any publication bias (t = 2.08, df = 5, *p* = 0.092), Supplementary Fig. 8.

### Change in HDL

Two studies contributed data on changes in HDL. Overall, roxadustat was associated with a non-significant difference in HDL compared with control (MD -0.10, 95% CI -0.20 to 0.00; *p* = 0.05; I² = 69%), Fig. 4C. A DOI plot was constructed to assess publication bias, and the LFK index of − 1.81 indicated minor asymmetry, suggesting potential publication bias, Supplementary Fig. 9.

### Change in Triglycerides

Three studies contributed data on changes in Triglycerides, reported at 7 time points. At 12 weeks, three studies showed a non-significant difference between roxadustat and control (MD -0.11, 95% CI -0.35 to 0.12; *p* = 0.35; I² = 0%). At 24 weeks, two studies demonstrated a non-significant effect (MD -0.07, 95% CI -0.34 to 0.19; *p* = 0.60; I² = 0%). At 40–48 weeks, two studies demonstrated a non-significant effect (MD -0.06, 95% CI -0.35 to 0.22; *p* = 0.66; I² = 2%). Overall, roxadustat was associated with a decrease in Triglycerides compared with control (MD -0.09, 95% CI -0.24 to 0.06; *p* = 0.26; I² = 0%); however, the result was non-significant. There was no evidence of subgroup differences between (*p* = 0.82), Fig. 4D. The funnel plot appeared moderately asymmetrical, suggesting some evidence of publication bias. Egger’s regression test, however, confirmed the absence of any publication bias (t = − 2.33, df = 5, *p* = 0.067), Supplementary Fig. 10.

### Change in CRP

**Fig. 5:**
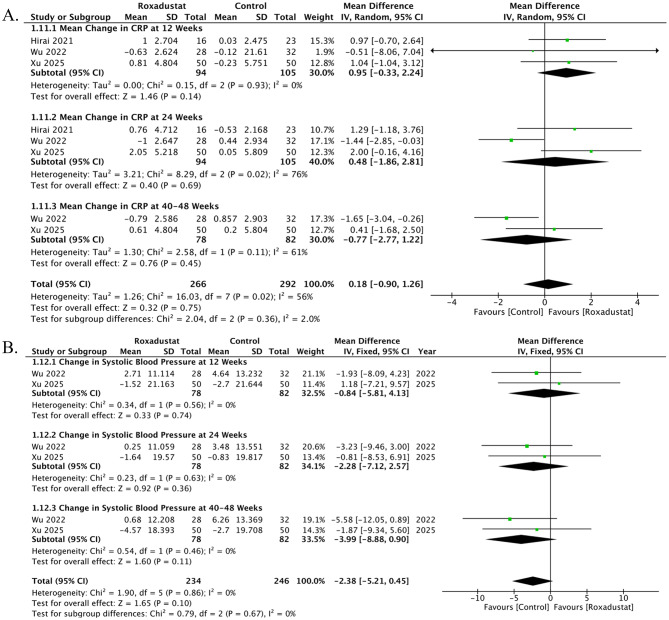

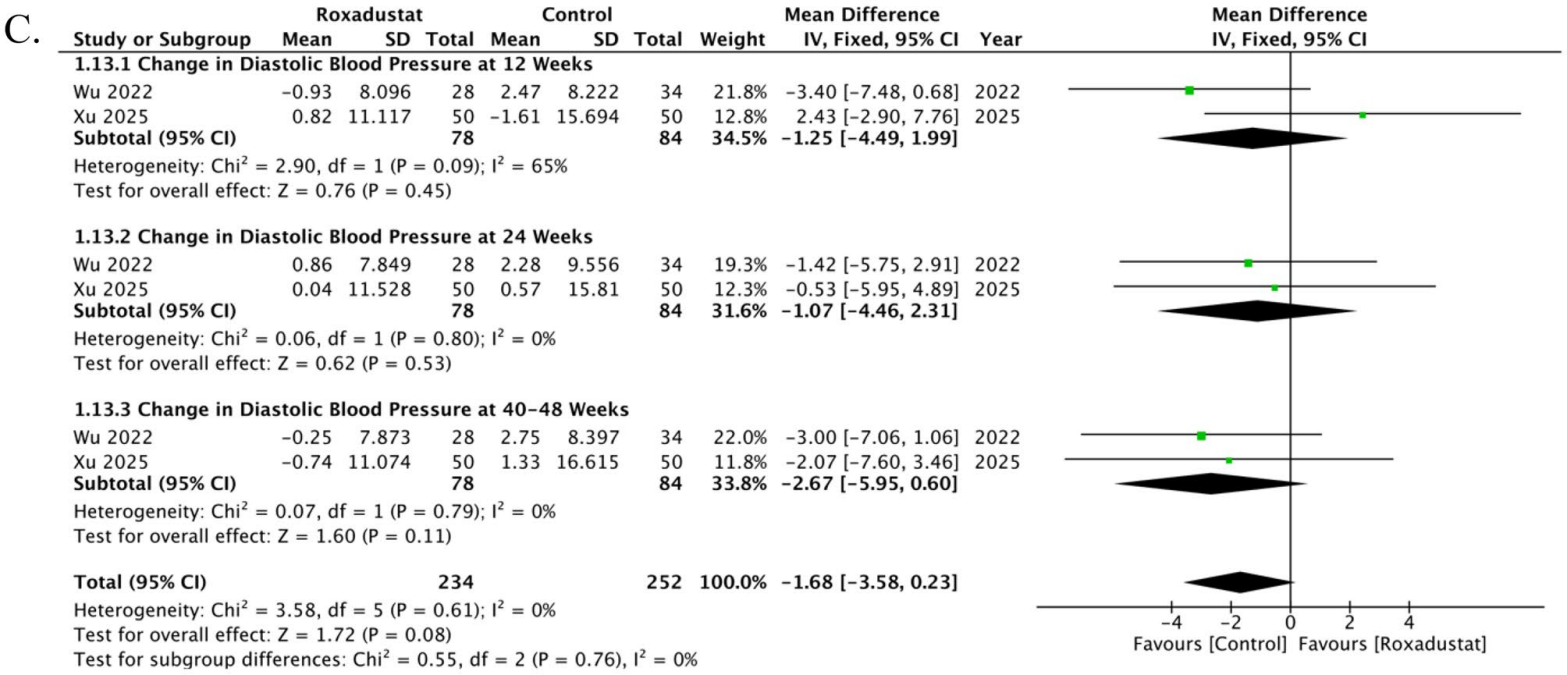
Effects of roxadustat versus control on inflammation and blood pressure parameters in peritoneal dialysis patients. (**A**) Change in C-reactive protein (CRP): Three studies at multiple time points showed no significant differences, with the overall effect indicating a non-significant reduction in CRP. (**B**) Change in systolic blood pressure (SBP): Two studies consistently reported non-significant reductions in SBP with roxadustat across follow-up periods. (**C**) Change in diastolic blood pressure (DBP): Two studies demonstrated a trend toward reduction in DBP, but the overall effect was non-significant

Three studies contributed data on changes in CRP, reported at 8 time points. At 12 weeks, three studies showed a non-significant difference between roxadustat and control (MD -0.95, 95% CI -0.33 to 2.24; *p* = 0.14; I² = 0%). At 24 weeks, three studies demonstrated a non-significant effect (MD -0.48, 95% CI -1.86 to 2.81; *p* = 0.69; I² = 76%). At 40–48 weeks, two studies demonstrated a non-significant effect (MD -0.77, 95% CI -2.77 to 1.22; *p* = 0.45; I² = 61%). Overall, roxadustat was associated with a decrease in CRP compared with control (MD 0.18, 95% CI -0.90 to 1.26; *p* = 0.75; I² = 56%); however, the result was non-significant. There was no evidence of subgroup differences between (*p* = 0.08), Fig. 5A. The funnel plot appeared moderately asymmetrical, suggesting some evidence of publication bias. Egger’s regression test, however, confirmed the absence of any publication bias t = 1.33, df = 6, *p* = 0.231, Supplementary Fig. 11.

#### Change in Systolic Blood Pressure

Two studies contributed data on changes in SBP, reported at 6 time points. At 12 weeks, two studies showed a non-significant difference between roxadustat and control (MD -0.84, 95% CI -5.81 to 4.13; *p* = 0.74; I² = 0%). At 24 weeks, two studies demonstrated a non-significant effect (MD -2.28, 95% CI -7.12 to 2.57; *p* = 0.36; I² = 0%). At 40–48 weeks, two studies demonstrated a non-significant effect (MD -3.99, 95% CI -8.88 to 0.90; *p* = 0.11; I² = 0%). Overall, roxadustat was associated with a decrease in SBP compared with control (MD -2.38, 95% CI -5.21 to 0.45; *p* = 0.10; I² = 0%); however, the result was non-significant. There was no evidence of subgroup differences between (*p* = 0.67), Fig. 5B. The funnel plot appeared moderately asymmetrical, suggesting some evidence of publication bias. Egger’s regression test, however, confirmed the absence of any publication bias (t = 2.03, df = 4, *p* = 0.112), Supplementary Fig. 12.

### Change in Diastolic Blood Pressure

Two studies contributed data on changes in DBP, reported at 6 time points. At 12 weeks, two studies showed a non-significant difference between roxadustat and control (MD -1.25, 95% CI -4.49 to 1.99; *p* = 0.45; I² = 65%). At 24 weeks, two studies demonstrated a non-significant effect (MD -1.07, 95% CI -4.46 to 2.31; *p* = 0.53; I² = 0%). At 40–48 weeks, two studies demonstrated a non-significant effect (MD -2.67, 95% CI -5.95 to 0.60; *p* = 0.11; I² = 0%). Overall, roxadustat was associated with a decrease in DBP compared with control (MD -1.68, 95% CI -3.58 to 0.23; *p* = 0.08; I² = 0%); however, the result was non-significant. There was no evidence of subgroup differences between (*p* = 0.67), Fig. 5C. The funnel plot appeared moderately asymmetrical, suggesting some evidence of publication bias. Egger’s regression test, however, confirmed the absence of any publication bias (*t* = 1.92, df = 4, and *p* = 0.13), Supplementary Fig. 13.

## Discussion

In our systematic review and meta-analysis, roxadustat demonstrated a statistically significant improvement in hemoglobin compared with control treatments, with consistent effects observed across multiple follow-up intervals. Although most iron biomarkers, inflammatory markers, blood pressure measures, and lipid parameters did not differ significantly from ESAs, our pooled results did reveal significant improvements in serum iron, TIBC, and hepcidin. The overall safety profile remains an area requiring further clarification, given the variability reported across studies. Below, we contextualize these findings in relation to previous reports focusing specifically on PD populations.

Our pooled analysis demonstrated a statistically significant rise in hemoglobin with roxadustat compared with control. This effect was consistent across timepoints, including 4, 8, 12, 16, and 24 weeks, with the most pronounced increases observed early in therapy. These findings are concordant with the trial by [9], which showed that roxadustat was noninferior to epoetin alfa, with mean hemoglobin increases of 0.7 g/dL versus 0.5 g/dL, respectively, and responder rates exceeding 87% in both groups by weeks 23–27. Similarly [25], reported marked hemoglobin increases among ESA-naïve PD patients (+ 1.69 g/dL) and modest rises in ESA-converted patients (+ 0.14 g/dL), suggesting that prior ESA exposure and baseline CKD-related anemia status may influence treatment responsiveness [20]. further demonstrated substantial improvements in hemoglobin among roxadustat-treated PD patients, from a baseline of 89.8 g/L to 118 g/L at week 16, while the ESA group showed no significant change. In contrast [18], reported long-term equivalence between roxadustat and rHuEPO, with both groups maintaining mean hemoglobin levels around 115 g/L and high rates of hemoglobin control over 24 months.

Beyond hemoglobin, our review revealed that roxadustat positively influenced iron metabolism, with pooled results showing increases in serum iron and total iron-binding capacity (TIBC), along with a significant reduction in hepcidin. These findings are consistent with the mechanistic role of HIF-PHIs in enhancing iron availability and mobilization, thereby reducing the need for intravenous iron supplementation [27]. A study by Chen et al. [9] provided compelling evidence in this domain, reporting a significant decline in hepcidin levels (− 30.2 ng/mL at week 27 with roxadustat versus − 2.3 ng/mL with epoetin alfa) and improvements in transferrin and TIBC. Akizawa et al. [25] also observed early reductions in hepcidin and improvements in transferrin, particularly during the initial four weeks of therapy. A meta-analysis [28], which included 19 studies, confirmed that roxadustat was associated with greater increases in serum iron, transferrin, TIBC, and transferrin saturation (TSAT) compared with ESAs in hemodialysis patients. These consistent findings across trials reinforce the advantage of roxadustat in optimizing iron utilization in PD patients, an important clinical consideration given the frequent challenges of maintaining adequate iron status in this population.

The use of roxadustat in the PD population warrants distinction from its use in HD. While the core mechanistic benefits—overcoming inflammation and reducing hepcidin—apply to all dialysis patients, roxadustat’s oral administration offers superior logistical convenience for home-based PD care, easing the burden of parenteral ESA injections and the need for frequent clinical visits for intravenous iron, a contrast to in-center HD management.

Additionally, our findings on the efficacy and safety of roxadustat in PD patients align closely with and are supported by a recent, large-scale pooled analysis of the four major Phase 3 trials (PYRENEES, SIERRAS, HIMALAYAS, and ROCKIES) [29–32]. This pooled analysis by Fliser et al. (2024) demonstrated that roxadustat was noninferior to ESAs in achieving and maintaining target hemoglobin levels in anemic PD patients [33]. Importantly, the analysis highlighted key advantages consistent with the HIF-PH inhibitor mechanism: roxadustat achieved noninferior efficacy with a stable mean weekly dose over time, whereas the mean weekly ESA dose increased by 24%. Furthermore, roxadustat-treated patients required significantly fewer intravenous iron infusions and were less likely to need a red blood cell (RBC) transfusion than ESA-treated patients. This consistent efficacy and superior iron management profile, particularly evident in the Phase 3 trials, reinforces our overall conclusion regarding roxadustat’s efficacy.

The unique advantage of roxadustat in PD is its ability to overcome ESA hyporesponsiveness (affecting 10%-20% of patients), which is often driven by inflammation and functional iron deficiency [33, 34]. As a HIF-PHI, roxadustat bypasses this barrier by stimulating endogenous erythropoietin and significantly reducing hepcidin. This superior iron utilization, demonstrated by increased serum iron and TIBC in our analysis, leads to fewer intravenous iron infusions. This, combined with its oral administration, makes it a vital alternative for PD patients resistant to standard ESA therapy.

Another area of interest is the potential metabolic benefits of roxadustat, particularly in lipid regulation. Our analysis showed small, non-significant reductions in total cholesterol, LDL, and triglycerides, although the direction of effect was consistently favorable. By comparison [9], demonstrated more robust lipid-lowering effects, with total cholesterol reduced by 0.69 mmol/L and LDL cholesterol by 0.62 mmol/L at week 27, alongside improvements in the LDL: HDL ratio. Similarly [20], reported that roxadustat was associated with significant reductions in total cholesterol and LDL cholesterol at weeks 12–16, and notably, these improvements were observed in both statin users and non-users. This observation is particularly relevant in the dialysis population, where reducing cholesterol and LDL is clinically meaningful. Previous studies have reported that lipid-lowering therapies are associated with reductions in all-cause mortality and MACE in patients with established ASCVD undergoing dialysis [35, 36]. Therefore, the ability of roxadustat to lower cholesterol and LDL, even in non-statin users, suggests that it may provide additional lipid-lowering benefits independent of statin use, potentially contributing to cardiovascular risk reduction in this high-risk group.

The interaction between inflammation and CKD-related anemia treatment efficacy is another important consideration. Conventional ESAs often show diminished efficacy in the setting of elevated inflammatory markers, which are common in dialysis patients. In contrast, roxadustat appears to maintain its effectiveness irrespective of inflammation. A previous study reported that hemoglobin levels in roxadustat-treated patients remained comparable regardless of C-reactive protein (CRP) status [9]. Additionally [20], demonstrated that roxadustat effectively corrected CKD-related anemia in patients with high inflammatory markers, whereas ESA therapy was less effective. Our meta-analysis found no significant differences in pooled CRP levels between treatment groups, though the broader meta-analysis [28] suggested that roxadustat reduces CRP levels across studies. These findings collectively support the view that roxadustat is a valuable option for PD patients with concurrent inflammation, a group often resistant to standard ESA therapy.

Cardiovascular safety is a key concern in evaluating any CKD-related anemia therapy. Our pooled analysis did not detect significant differences in systolic or diastolic blood pressure between treatment groups, although there was a numerical trend toward lower blood pressure with roxadustat. More compelling evidence that patients treated with rHuEPO experienced significant increases in blood pressure and higher rates of nocturnal hypertension over 24 months, whereas no such changes were observed in the roxadustat group [28]. Furthermore, reporting a higher incidence of cardiovascular and cerebrovascular complications in the rHuEPO group compared with the roxadustat group (26.7% vs. 10%), with multivariable analysis identifying roxadustat as a protective factor [28]. These findings suggest a potential for roxadustat to influence long-term cardiovascular outcomes compared with ESAs; however, the overall cardiovascular benefit remains a hypothesis that requires confirmation in large-scale randomized controlled trials.

Several factors may explain the discrepancies observed across studies. Differences in patient populations, such as ESA-naïve versus ESA-converted patients, can substantially affect hemoglobin responses. Variations in dosing regimens, titration protocols, and adherence may also contribute to heterogeneity. Iron management strategies differ across centers, with some studies favoring intravenous supplementation and others relying more on oral iron. While oral iron is often insufficient in ESA-treated HD patients due to ongoing blood losses and inflammation, it may be partly effective in PD patients, where such losses are minimal; these differences could influence iron markers such as ferritin and TSAT. Additionally, the duration of follow-up plays a crucial role in determining metabolic and cardiovascular outcomes.

Because our meta-analysis pooled studies with different ESA exposure histories (ESA-naïve, ESA-converted, and mixed cohorts), the summary estimates should be interpreted as reflecting the average effect of a “roxadustat-based strategy” across these heterogeneous clinical scenarios, rather than providing separate, robust estimates for ESA-naïve and ESA-converted patients.

### Strengths and limitations

Our study has several strengths, including a focus on PD populations, a comprehensive assessment of multiple relevant outcomes, and timepoint-specific evaluation of hemoglobin changes. Nevertheless, limitations must be acknowledged. Heterogeneity across trials, potential publication bias, and the relatively small sample sizes of PD-specific studies constrain the generalizability of our findings. In addition, the predominance of retrospective cohort designs and variability in study quality further limit the robustness of the pooled estimates. Although these designs reflect the current evidence base in PD populations, they inherently carry risks of residual confounding and incomplete data capture. Moreover, all included studies were conducted in China or Japan, which may restrict the external validity of our findings. Differences in clinical practice patterns, regulatory environments, and population characteristics outside East Asia should be considered when interpreting and applying these results.

Future research should prioritize large randomized controlled trials in PD patients with sufficient power to assess cardiovascular and thromboembolic outcomes, ideally with standardized iron management protocols and longer follow-up durations. Stratification by ESA-naïve versus ESA-converted status and by inflammatory burden would provide further clarity on which subgroups benefit most from roxadustat. Additionally, studies exploring the lipid-lowering and anti-inflammatory effects of roxadustat could help elucidate its potential cardiovascular benefits.

Finally, due to the design of our meta-analysis, we could not account for concomitant iron supplementation or variations in HIF-PHI dosing over time. Notably, higher doses reported in the literature (100–120 mg three times per week) may be excessive for many patients, potentially leading to overcorrection of anemia or polycythemia and an increased risk of adverse events.

## Conclusion

In patients undergoing peritoneal dialysis, roxadustat demonstrates clinically meaningful efficacy in correcting anemia, achieving consistent and significant improvements in hemoglobin across all evaluated time points. The drug also enhances iron metabolism, evidenced by increased serum iron and TIBC and reduced hepcidin. However, roxadustat shows no clear advantage regarding ferritin, TSAT, lipid profiles, inflammatory markers, or blood pressure, and thus its broader metabolic or cardiovascular benefits remain unproven in the PD population. Based on current evidence, roxadustat represents an effective oral alternative for anemia management in PD patients. Larger and longer-term trials are needed to establish its safety profile and to validate our findings in routine clinical practice.

## Supplementary Information

Below is the link to the electronic supplementary material.

Supplementary Material 1

## Data Availability

Data are available upon reasonable request from the corresponding author.

## Abbreviations

ASCVD: Atherosclerotic cardiovascular disease
BMI: Body mass index
CI: Confidence interval
CRP: C–reactive protein
DBP: Diastolic blood pressure
DOI: Difference of means plot (for publication bias assessment)
ESA: Erythropoiesis–stimulating agent
Hb: Hemoglobin
HDL: High–density lipoprotein
HIF: Hypoxia–inducible factor
HIF: PHI–Hypoxia–inducible factor prolyl hydroxylase inhibitor
I²: Inconsistency index (heterogeneity statistic)
LDL: Low–density lipoprotein
LFK: Luis Furuya–Kanamori index (publication bias metric)
MACE: Major adverse cardiovascular events
MD: Mean difference
PD: Peritoneal dialysis
PRISMA: Preferred Reporting Items for Systematic Reviews and Meta–Analyses
RCT: Randomized controlled trial
RevMan: Review Manager software
rHuEPO: Recombinant human erythropoietin
ROBINS: I–Risk of Bias in Non–randomized Studies of Interventions
RoB 2: Risk of Bias 2 tool
SBP: Systolic blood pressure
TIBC: Total iron–binding capacity
TSAT: Transferrin saturation
WOS: Web of Science
