# Supplementary material for: Roxadustat for CKD-related anemia in patients undergoing peritoneal dialysis: a systematic review and meta-analysis
Source: BMC Nephrol. 2026 May 25;27:340. doi: 10.1186/s12882-025-04723-x (PMC13214376; doi:10.1186/s12882-025-04723-x)
Supplement: Supplementary file 1 — Supplementary Material 1 [file 12882_2025_4723_MOESM1_ESM.docx]

| S****upplementary Table 1. search strategy**** | | | |
| --- | --- | --- | --- |
| Database | **Date of search** | **Search Terms** | **Results (n)** |
| PubMed | 20th July 2025 | (Roxadustat OR roxadustat OR FG-4592) AND ("Peritoneal Dialysis"[MeSH] OR peritoneal dialysis OR CAPD OR CCPD) AND ("Anemia"[MeSH] OR anemia OR anaemia OR "Iron Metabolism"[MeSH] OR “iron metabolism” OR “iron parameters” OR ferritin OR transferrin OR hepcidin) | 35 |
| Scopus | 20th July 2025 | TITLE-ABS-KEY ( (Roxadustat OR roxadustat OR FG-4592) AND ("Peritoneal Dialysis" OR peritoneal dialysis OR CAPD OR CCPD) AND ("Anemia" OR anemia OR anaemia OR "Iron Metabolism" OR "iron metabolism" OR "iron parameters" OR ferritin OR transferrin OR hepcidin) ) | 78 |
| Web of Science (WOS) | 20th July 2025 | (Roxadustat OR roxadustat OR FG-4592) AND ("Peritoneal Dialysis" OR peritoneal dialysis OR CAPD OR CCPD) AND ("Anemia" OR anemia OR anaemia OR "Iron Metabolism" OR "iron metabolism" OR "iron parameters" OR ferritin OR transferrin OR hepcidin) | 46 |

| **Supplementary Table 2. Baseline characteristics of the studies included in the systematic review and meta-analysis** | | | | | | | | | | | | | | | | | | | | | | | | | | | | | | | | | | | | | | |
| --- | --- | --- | --- | --- | --- | --- | --- | --- | --- | --- | --- | --- | --- | --- | --- | --- | --- | --- | --- | --- | --- | --- | --- | --- | --- | --- | --- | --- | --- | --- | --- | --- | --- | --- | --- | --- | --- | --- |
| Study and publication year | Age | | Sex (M) | | BMI | | Diabetes mellitus | | Hypertension | | Duration of dialysis (mo.) | | Systolic blood pressure  (mmHg) | | Diastolic blood pressure  (mmHg) | | Hemoglobin (g/L) | | Ferritin  (ng/mL) | | Iron (μg/dL) | | Transferrin saturation (%) | | Serum creatinine | | Blood urea nitrogen | | Albumin (g/L) | | Total cholesterol (mmol/L) | | Triglycerides(mmol/L) | | LDL  (mmol/L) | | HDL  (mmol/L) | |
|  | Roxadustat | Control | Roxadustat | Control | Roxadustat | Control | Roxadustat | Control | Roxadustat | Control | Roxadustat | Control | Roxadustat | Control | Roxadustat | Control | Roxadustat | Control | Roxadustat | Control | Roxadustat | Control | Roxadustat | Control | Roxadustat | Control | Roxadustat | Control | Roxadustat | Control | Roxadustat | Control | Roxadustat | Control | Roxadustat | Control | Roxadustat | Control |
| Akizawa 2020 | 66.1 (10.9) |  | 76.90% |  | 23.10 (1.67) |  |  |  |  |  | 36.45 (37.86) |  |  |  |  |  | 9.35 (0.75) |  | 269.57 (130.30) |  | 102.8 (25.6 |  | 45.76 (14.46) |  |  |  |  |  |  |  |  |  |  |  |  |  |  |  |
| Bao 2022 | 51.6 ± 13.4 |  | 55.20% |  | 22.18 ± 2.61 |  |  |  |  |  |  |  | 143 ± 21 |  | 81 ± 12 |  | 9.51 ± 1.01 |  |  |  | 12.9 ± 4.7 |  |  |  |  |  |  |  |  |  |  |  |  |  |  |  |  |  |
| Cheng 2023 | 46.6 ± 10.8 | 49.3 ± 11.2 | 29 (48.3%) | 29 (48.3%) |  |  | 6 (10%) | 7 (11.7%) | 55 (91.6%) | 54 (90%) | 10 ± 17.85 | 11.27 ± 18.84 | Mean arterial pressure | Mean arterial pressure | Mean arterial pressure | Mean arterial pressure | 8.96 ± 1.37 | 9.02 ± 1.35 | NR | NR | NR | NR | NR | NR | weekly creatinine clearance (L/1.73m2) | | Kt/V, weekly urea  clearance index | | 37.6 ± 4.4 | 38.7 ± 4.6 | 4.5 ± 1.1 | 4.6 ± 1.1 | 1.7 ± 0.9 | 1.8 ± 1.0 | 2.5 ± 0.7 | 2.5 ± 0.8 | 1.1 ± 0.3 | 1.2 ± 0.4 |
| Hirai 2021 | 58.0 ± 13.8 | 62.1 ± 13.9 | 7 (43.8%) | 16 (69.6%) | 20.7 ± 3.3 | 24.5 ± 4.1 | 4 (25.0%) | 8 (34.8%) | NR | NR | 51.4 ± 62.9 | 29.83 ± 36.74 | 136.8 ± 15.3 | 145.2 ± 19.7 | 81.4 ± 13.4 | 81.1 ± 17.4 | 10.7 ± 1.2 | 10.5 ± 1.3 | 145.1 ± 129.6 | 180.7 ± 88.7 | NR | NR | 32.6 ± 9.2 | 40.9 ± 12.9 | 32.6 ± 9.3 (mg/dL) | 40.9 ± 12.10 (mg/dL) | 56.3 ± 10 (mg/dL) | 59.9 ± 14.1 (mg/dL) | 34 ± 3 | 34 ± 6 | NR | NR | NR | NR | NR | NR | NR | NR |
| Hou 2021 | 48 ± 12 | 48.3 ± 13 | 47 (54.7%) | 25 (58.1%) | 23.7 ± 3.5 | 24.1 ± 3.2 | diabetic nephropathy 12 (14%) | 8 (19%) | hypertensive nephropathy 29 (34%) | 14 (33%) | NR | NR | 148.4 ± 14.7 | 146.4 ± 10.4 | 92.5 ± 10.7 | 91.5 ± 7.0 | 9.0 ± 1.4 | 9.0 ± 1.2 | 268.8 ± 297.2 | 257.4 ± 190.8 | 12.6 ± 5.0 | 11.4 ± 5.0 | 31.3 ± 14.2 | 29.6 ± 13.2 | NR | NR | NR | NR | NR | NR | 4.7 ± 1.1 | 5.0 ± 1.5 | 1.7 ± 1.4 | 1.6 ± 0.6 | 2.6 ± 0.8 | 2.8 ± 0.9 | 1.1 ± 0.3 | 1.1 ± 0.4 |
| Liu 2024 | 57.1 ± 15.2 | 60.0 ± 11.3 | 40 (65.6%) | 12 (60%) | NR | NR | 22 (36.1%) | 8 (40%) | 48 (78.7%) | 17 (85%) | 18.0 ± 8.68 | 21.6 ± 8.98 | NR | NR | NR | NR | 8.98 ± 1.89 | 9.52 ± 1.60 | 318 ± 315 | 230 ± 130 | 10.5 ± 6.23 | 11.5 ± 8.67 | 24.8 ± 5.9 | 23.6 ± 4.23 | 764 ± 412 µmol/L | 685 ± 201 µmol/L | 27.6 ± 10.2 mmol/L | 28.6 ± 5.01 mmol/L | 36.3 ± 4.01 | 37.6 ± 3.77 | 4.56 ± 1.41 | 4.71 ± 1.20 | 2.13 ± 1.41 | 2.12 ± 1.45 | 3.46 ± 1.61 | 3.70 ± 1.09 | 1.40 ± 0.612 | 1.44 ± 0.503 |
| Liu 2025 | 55.6 ± 16.1 | 58.1 ± 13.4 | 23 (67.6%) | 72 (60%) | NR | NR | Diabetic nephropathy 9 (26.5%) | Diabetic nephropathy 37 (30.8%) | Hypertensive nephropathy 7 (20.6%) | Hypertensive nephropathy 25 (20.8%) | 34.1 ± 32.7 | 34.0 ± 40.2 | NR | NR | NR | NR | 11.0 ± 0.998 | 11.0 ± 1.66 | 259 ± 252 | 266 ± 266 | 10.4 ± 5.75 | 12.4 ± 8.88 | 23.2 ± 4.01 | 23.8 ± 5.10 | 706 ± 186 µmol/L | 787 ± 399 µmol/L | 27.4 ± 7.14 mmol/L | 27.7 ± 9.04 mmol/L | 36.2 ± 4.32 | 37.1 ± 4.49 | 4.29 ± 0.779 | 4.62 ± 1.19 | 2.21 ± 1.49 | 1.98 ± 1.28 | 3.71 ± 1.43 | 3.57 ± 1.46 | 1.36 ± 0.558 | 1.39 ± 0.571 |
| Wu 2022 | 39.71 ± 9.61 | 43.44 ± 11.89 | 17 (60.7%) | 18 (56.3%) | 23.79 ± 4.23 | 23.52 ± 3.56 | 3 (10.7%) | 2 (6.3%) | 24 (85.7%) | 26 (81.3%) | NR | NR | 133.68 ± 12.51 | 134.55 ± 14.96 | 83.39 ± 8.97 | 82.00 ± 7.78 | 8.13 ± 1.29 | 8.54 ± 1.47 | 2.33 ± 0.49 (log10) | 2.26 ± 0.43 (log10) | 14.37 ± 4.83 | 14.00 ± 3.57 | 34 ± 11 | 33 ± 10 | 980.27 ± 239.49 µmol/L | 960.20 ± 213.67 µmol/L | 36.31 ± 8.85 mmol/L | 36.23 ± 8.11 mmol/L | 39.68 ± 7.84 | 37.55 ± 4.17 | 3.67 ± 0.90 | 3.89 ± 1.22 | 1.18 ± 0.58 | 1.31 ± 0.48 | 2.24 ± 0.74 | 2.38 ± 0.85 | 0.97 ± 0.31 | 0.98 ± 0.29 |
| Xua 2025 | 51.56 ± 13.15 | 55.06 ± 13.46 | 28 (56%) | 29 (58%) | 23.70 ± 4.26 | 24.06 ± 3.13 | NR | NR | NR | NR | 26.5 (55.3) | 24.6 (49.6) | 142.64 ± 21.15 | 144.63 ± 21.11 | 84.31 ± 11.78 | 84.59 ± 15.12 | 9.493 ± 1.778 | 9.844 ± 1.540 | 160.4 ± (282.15) | 191.4 ± (208.8) | 15.19 ± 7.77 | 15.71 ± 6.25 | 32.7 (20.6) | 38.1 (20.3) | 881± 420 µmol/L | 877 ± 515 µmol/L | NR | NR | 33.37 ± 4.63 | 34.97 ± 5.04 | 4.30 ± 1.26 | 4.66 ± 1.37 | 1.77 ± 1.13 | 2.06 ± 1.33 | 2.65 ± 1.00 | 2.87 ± 1.09 | 1.00 ± 0.33 | 1.03 ± 0.38 |
| Zhang 2023 | 47.59 ± 13.63 | 50.15 ± 12.02 | 59 (55.66%) | 27 (50.94%) | 23.58 ± 3.66 | 24.80 ± 4.01 | History of DM 20(18.87) | History of DM 10(18.87) | History of HTN 94(88.68) | History of HTN 45(84.91) | 81.42 ± 60.69 | 112 ± 131.83 | NR | NR | NR | NR | 8.784 ± 0.942 | 8.666 ± 1.072 | 214.24 ± 212.69 | 211.48 ± 206.26 | 13.54 ± 6.46 | 12.80 ± 5.39 | 32.13 ± 15.23 | 31.98 ± 14.85 | 820.92 ± 216.48 µmol/L | 883.87 ± 302.90 µmol/L | NR | NR | NR | NR | 4.01 ± 1.20 | 4.23 ± 1.14 | 1.54 ± 0.85 | 1.78 ± 1.33 | 2.43 ± 1.00 | 2.53 ± 0.97 | 0.91 ± 0.32 | 0.93 ± 0.30 |
| Zhu 2021 |  |  | 13 (31.9%) |  |  |  |  |  |  |  |  |  |  |  |  |  | 86.2 ± 14.8 |  |  |  |  |  |  |  |  |  |  |  |  |  |  |  |  |  |  |  |  |  |


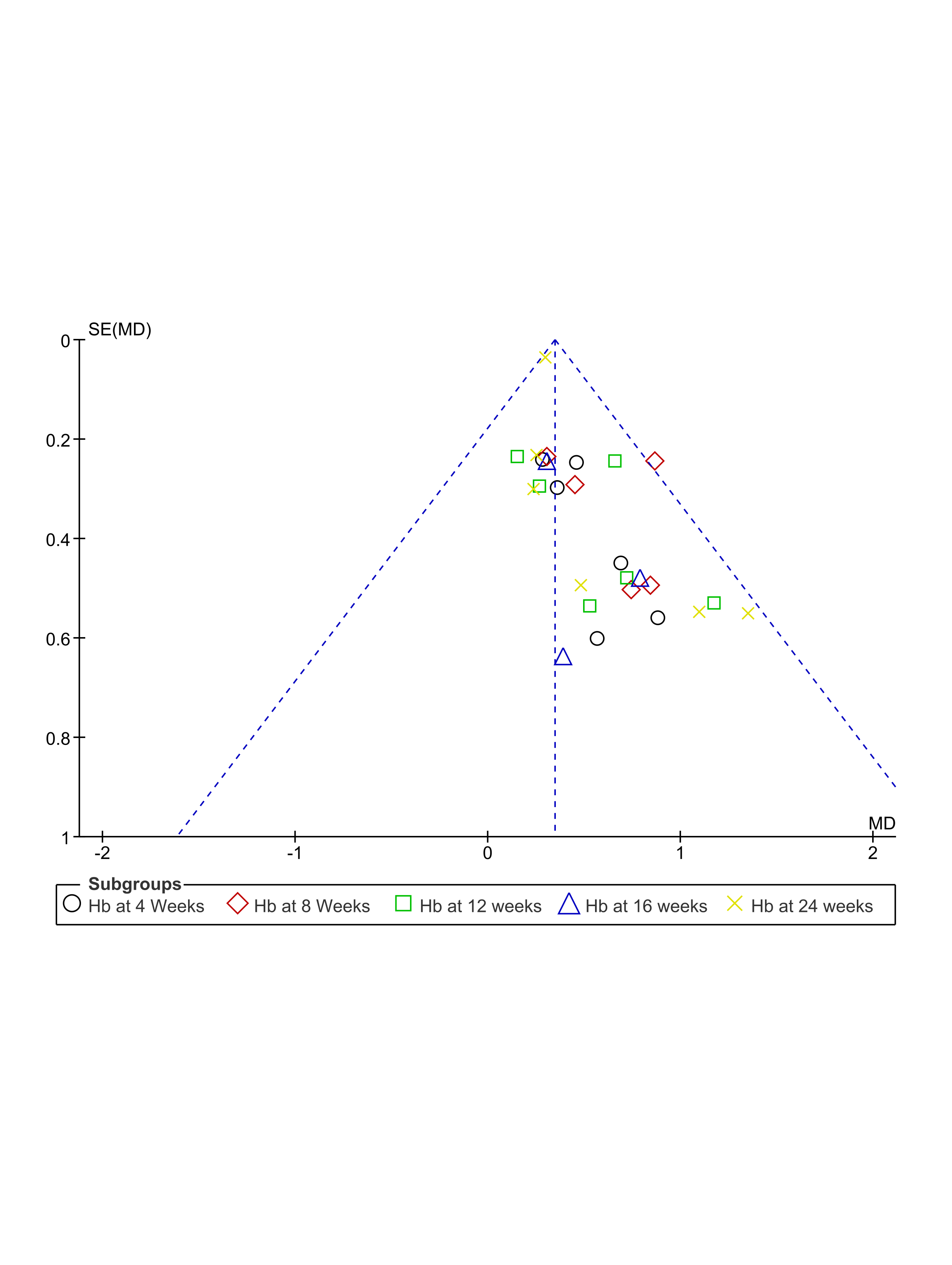


**Supplementary Figure 1.** Funnel plot assessing publication bias for the meta-analysis of hemoglobin (Hb) change with roxadustat versus control. Visual inspection suggested asymmetry, and Egger’s regression test confirmed the presence of publication bias (t = 4.36, df = 25, p = 0.0002).


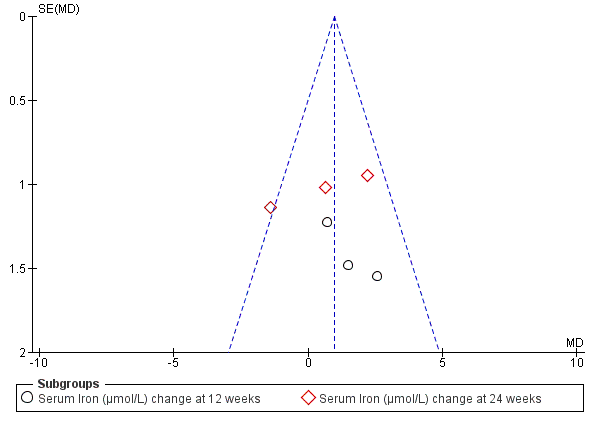


**Supplementary Figure 2.** Funnel plot for serum iron. The plot appeared symmetrical, and Egger’s regression test confirmed the absence of publication bias (t = 0.22, df = 4, p = 0.84).


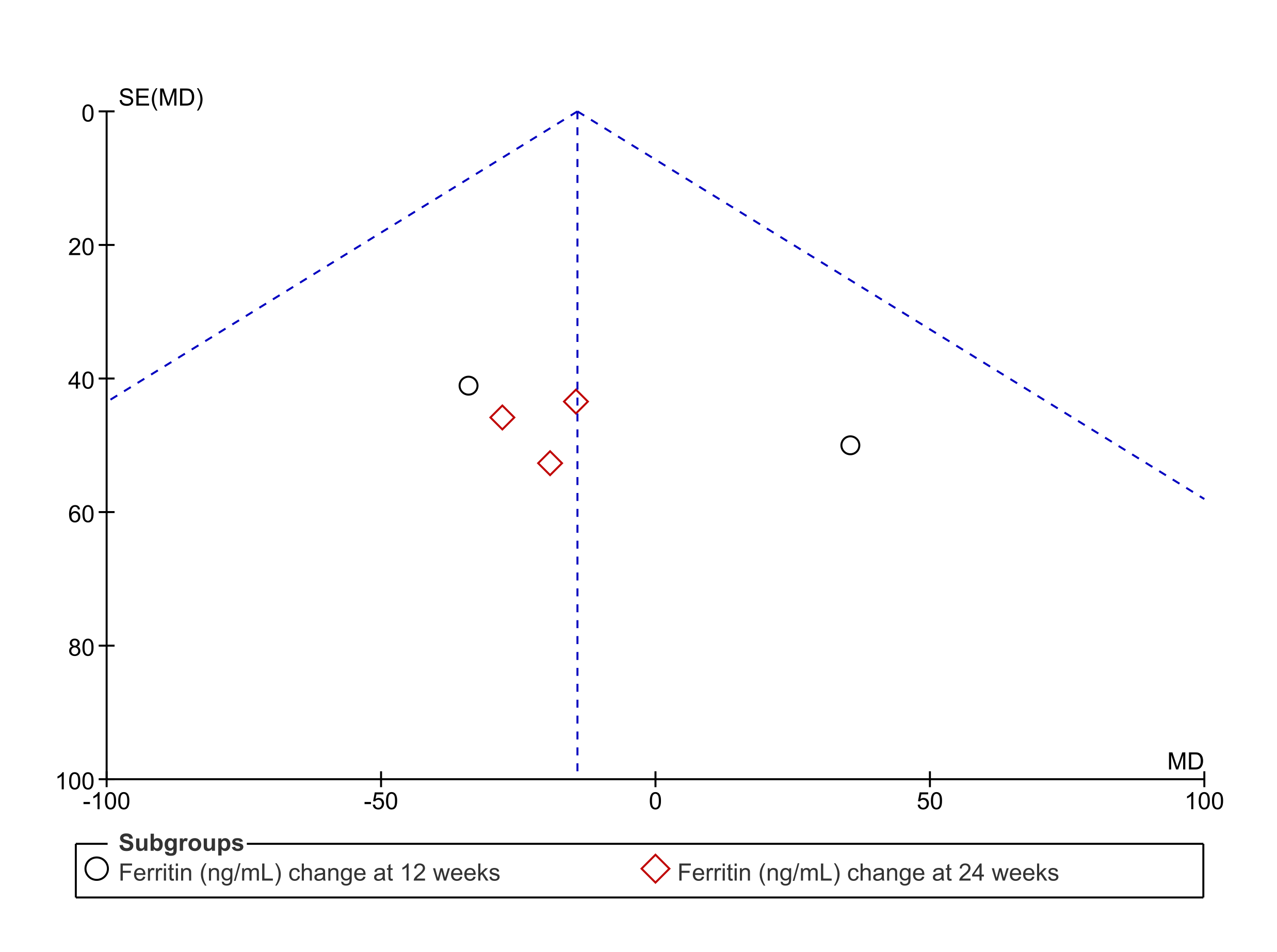


**Supplementary Figure 3.** Funnel plot for serum ferritin. The plot appeared slightly asymmetrical, suggesting potential publication bias; however, Egger’s regression test indicated no significant small-study effects (t = 1.14, df = 3, p = 0.34).


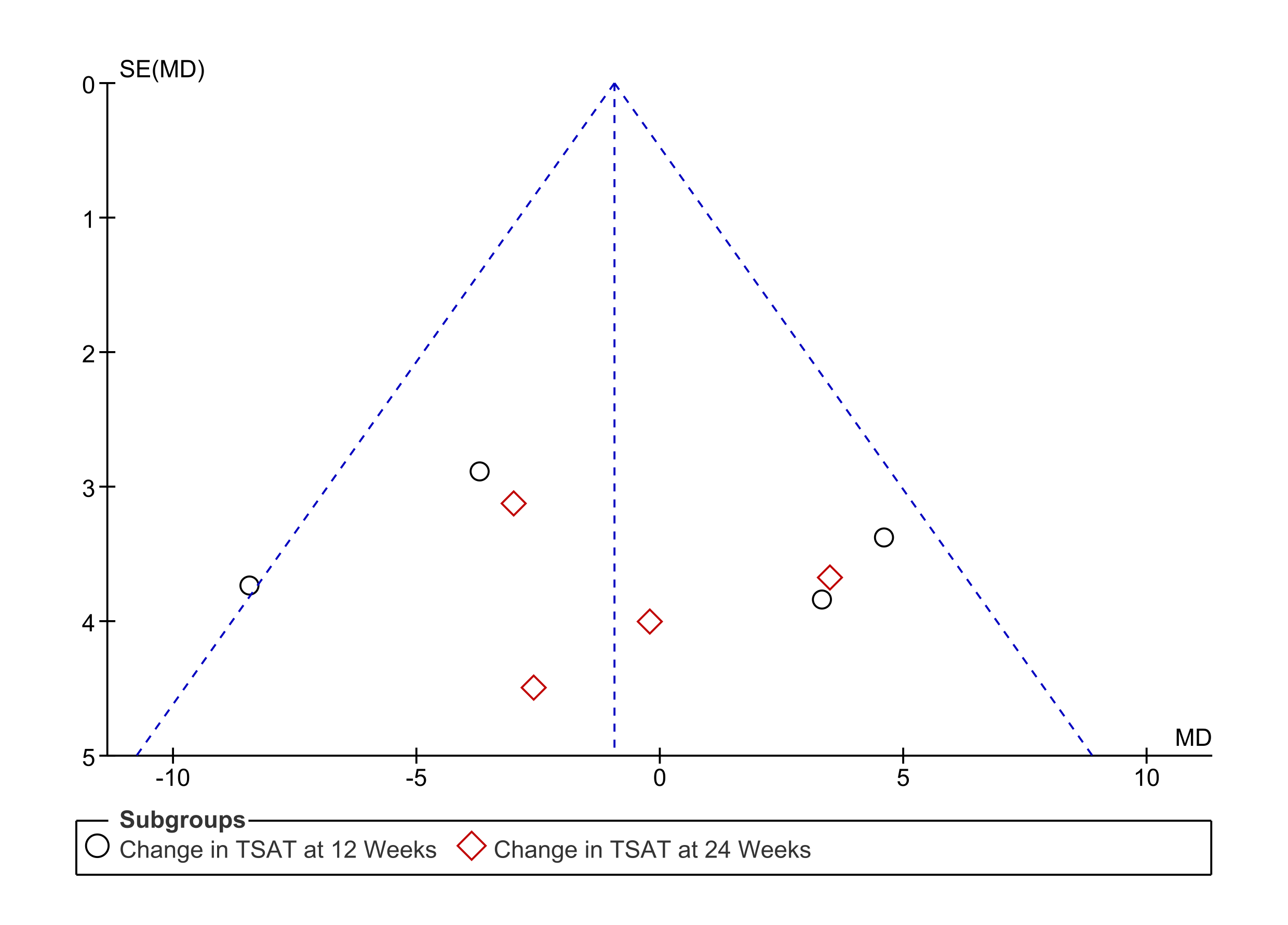


**Supplementary Figure 4.** Funnel plot for transferrin saturation (TSAT). The plot appeared symmetrical, and Egger’s regression test confirmed the absence of publication bias (t = 0.38, df = 6, p = 0.72).


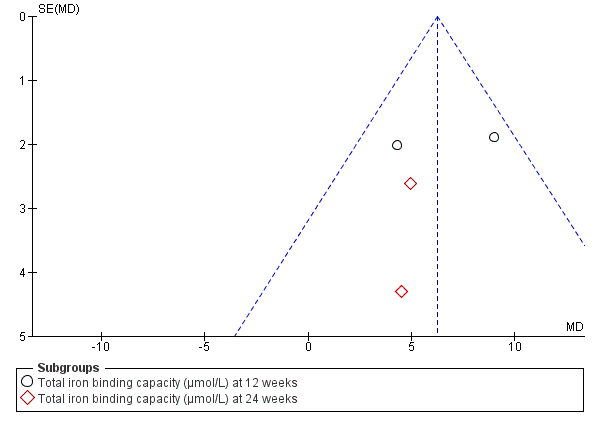


**Supplementary Figure 5.** Funnel plot for total iron-binding capacity (TIBC). The plot appeared symmetrical, and Egger’s regression test confirmed the absence of publication bias (t = –0.66, df = 2, p = 0.576).


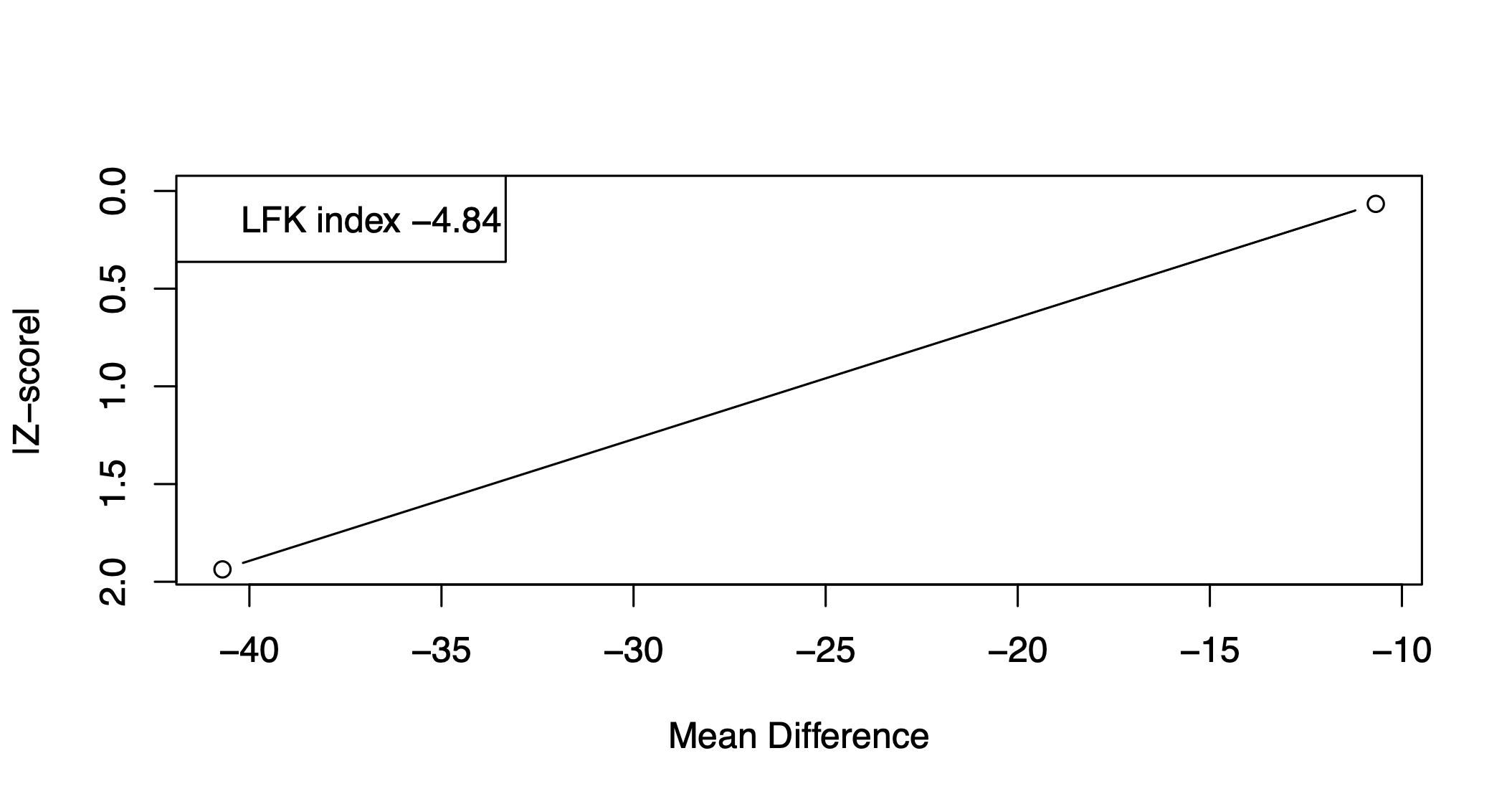


**Supplementary Figure 6.** DOI plot for hepcidin. The LFK index (–4.84) indicated major asymmetry, suggestive of significant publication bias.


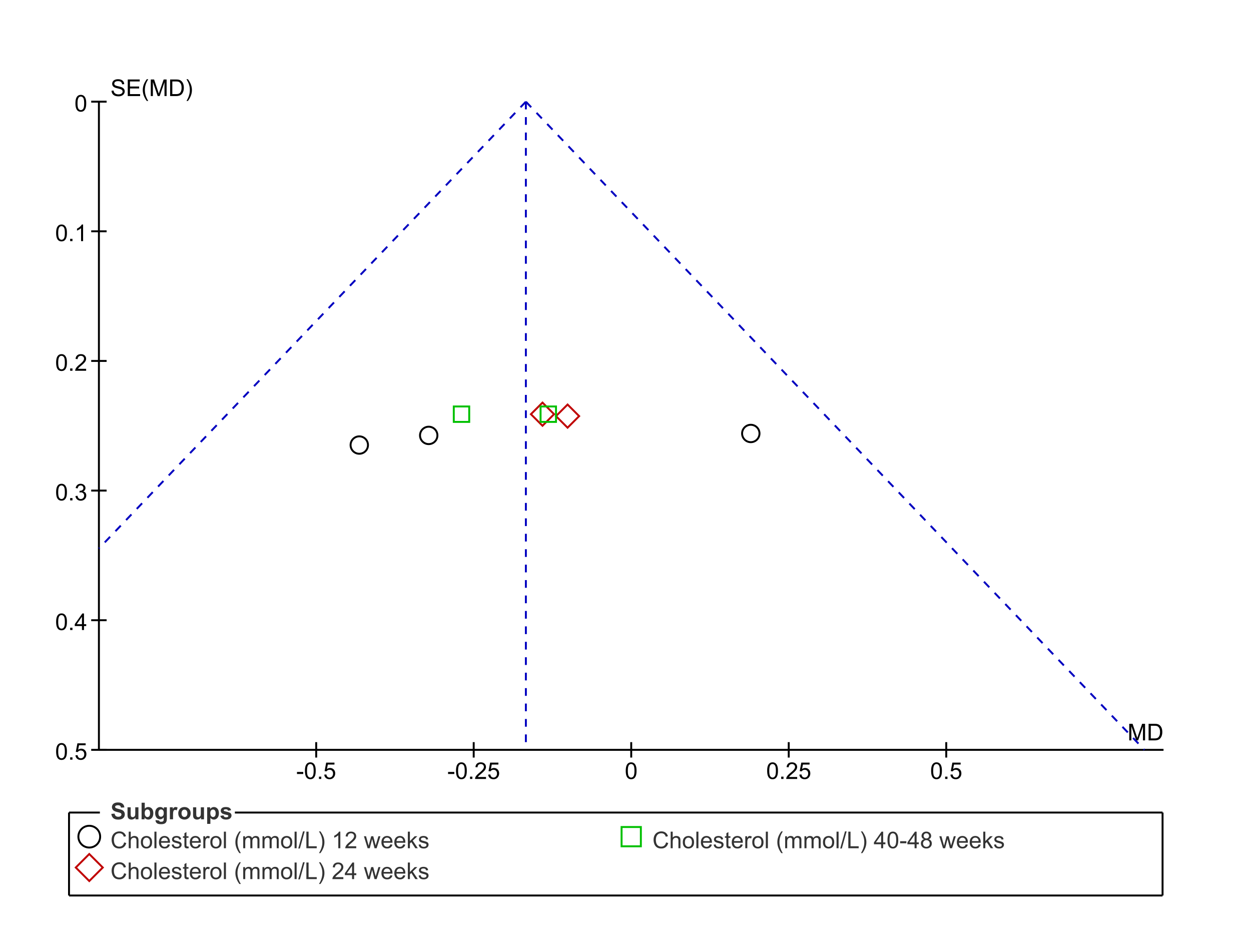


**Supplementary Figure 7.** Funnel plot for serum cholesterol. The plot appeared symmetrical, and Egger’s regression test indicated no evidence of small-study effects (t = –0.53, df = 5, p = 0.62).

**
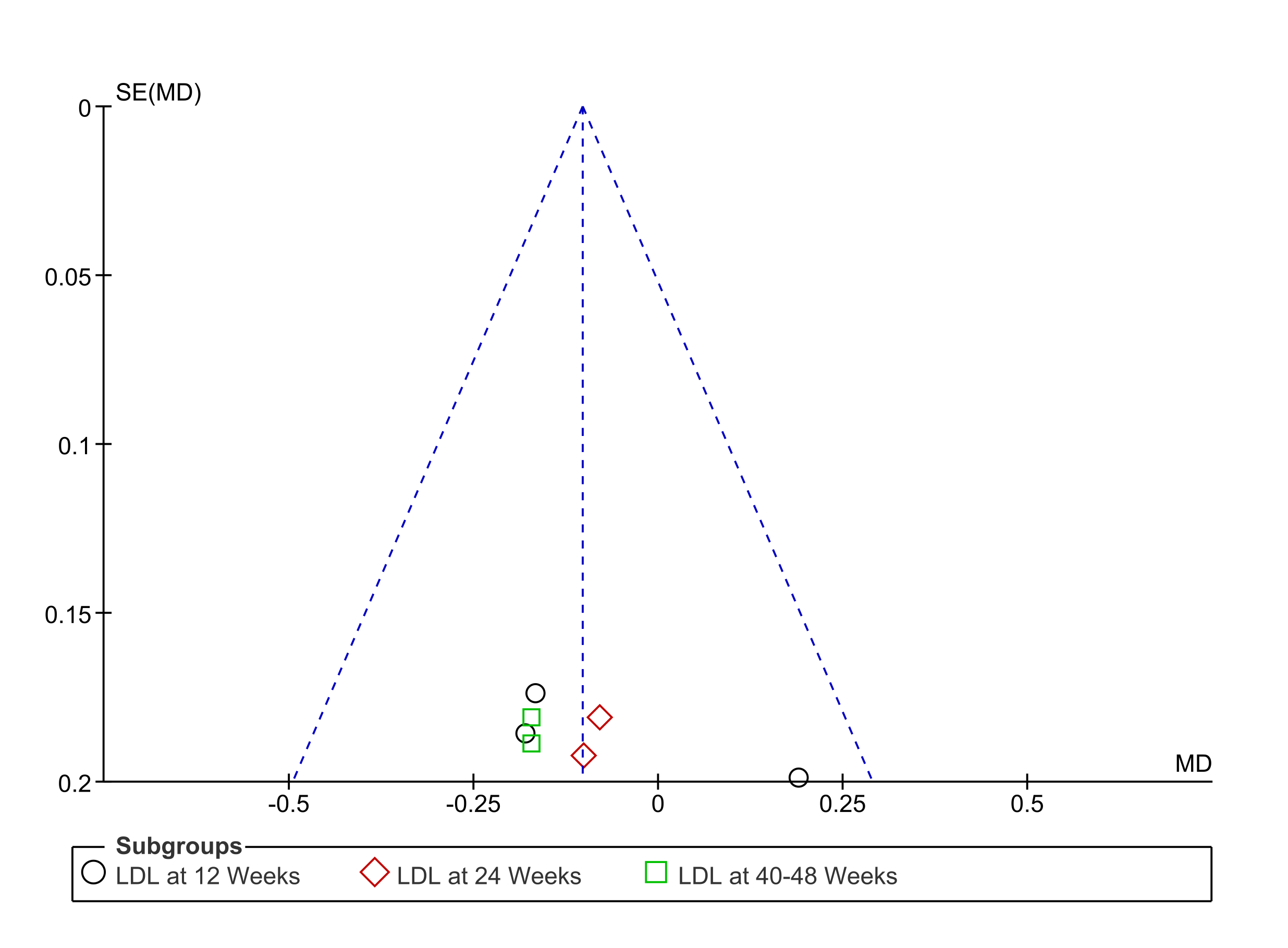
**

**Supplementary Figure 8.** Funnel plot for low-density lipoprotein (LDL). The plot appeared symmetrical, and Egger’s regression test confirmed the absence of publication bias (t = 2.08, df = 5, p = 0.092).


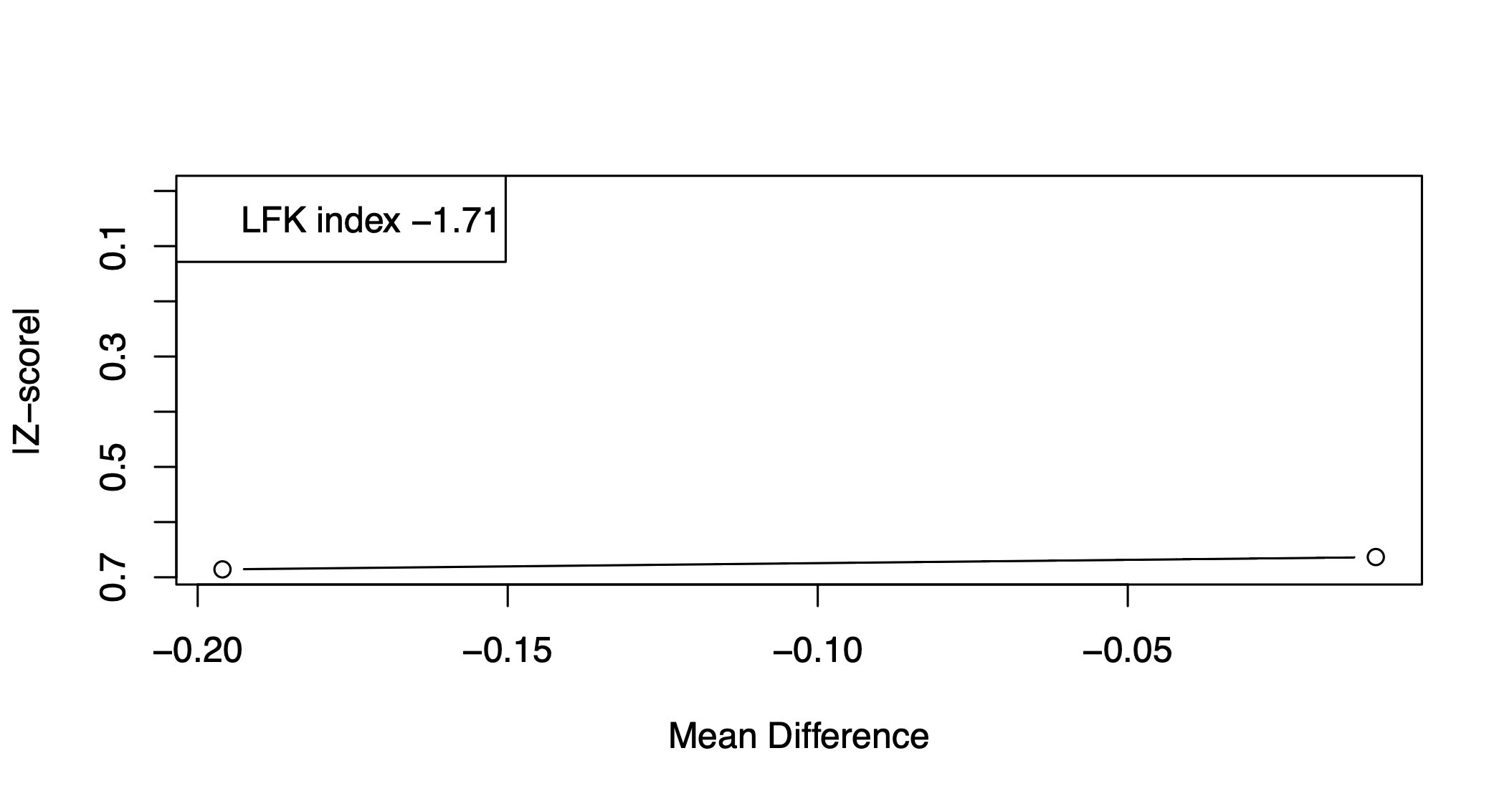


**Supplementary Figure 9.** DOI plot for high-density lipoprotein (HDL). The LFK index (–1.81) suggested minor asymmetry, consistent with potential publication bias.


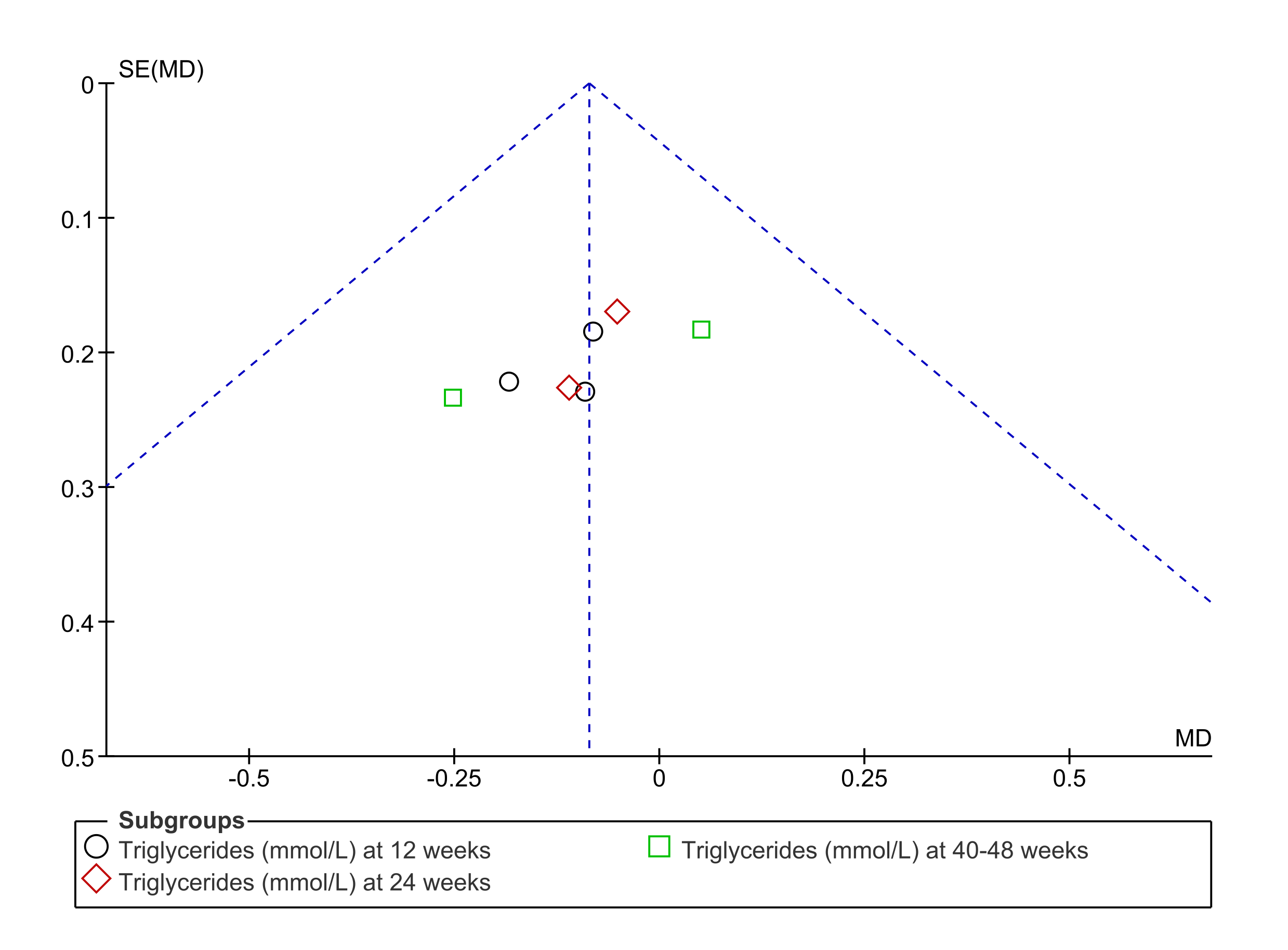


**Supplementary Figure 10.** Funnel plot for triglycerides. The plot appeared moderately asymmetrical, suggesting some evidence of publication bias; however, Egger’s regression test did not confirm statistical significance (t = –2.33, df = 5, p = 0.067).


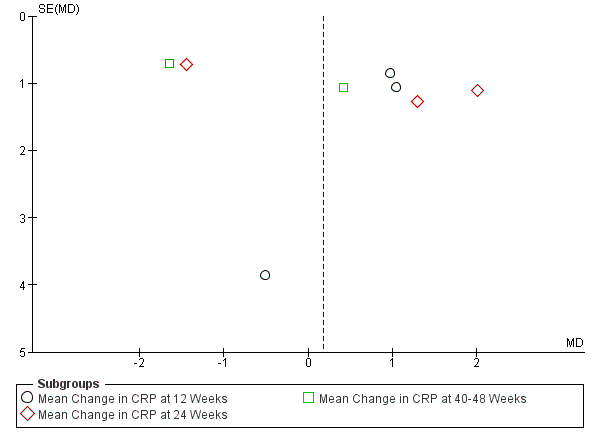


**Supplementary Figure 11.** Funnel plot for C-reactive protein (CRP). The plot appeared moderately asymmetrical, suggesting some evidence of publication bias; however, Egger’s regression test did not confirm statistical significance (t = 1.33, df = 6, p = 0.231).

**
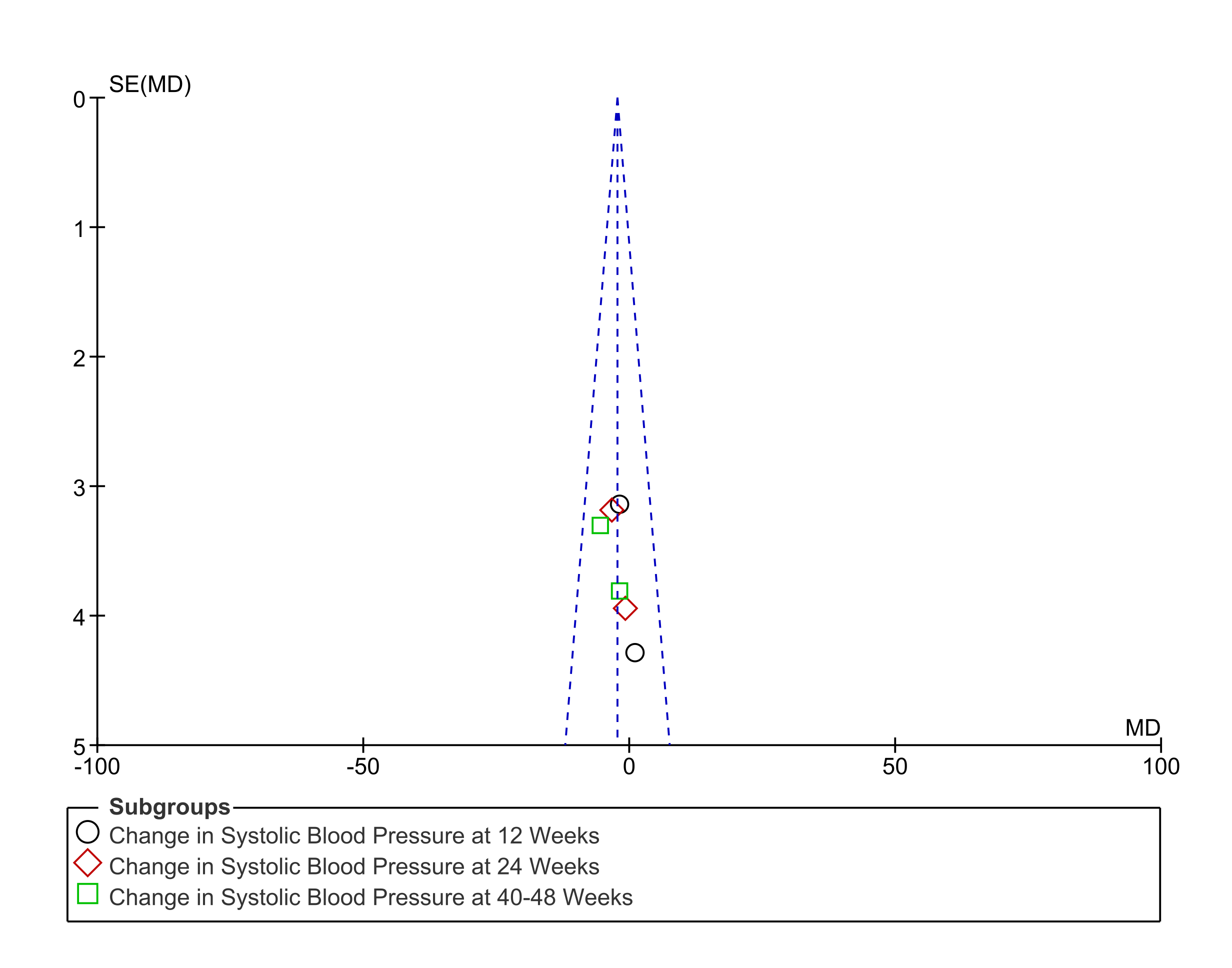
**

**Supplementary Figure 12.** Funnel plot for systolic blood pressure (SBP). The plot appeared moderately asymmetrical, suggesting some evidence of publication bias; however, Egger’s regression test indicated no significant bias (t = 2.03, df = 4, p = 0.112).


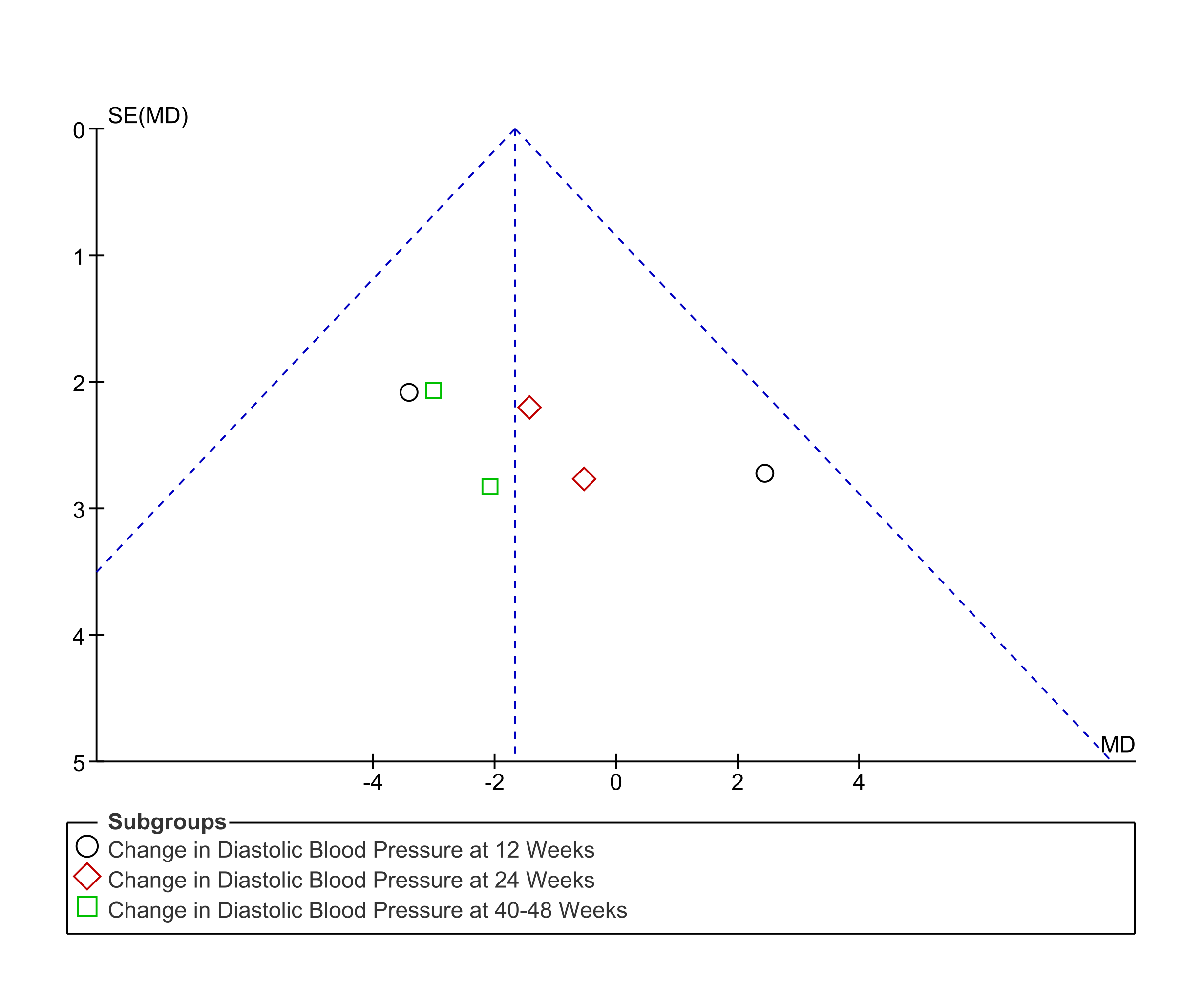


**Supplementary Figure 13.** Funnel plot for diastolic blood pressure (DBP). The plot appeared moderately asymmetrical, suggesting some evidence of publication bias; however, Egger’s regression test confirmed the absence of any publication bias (t = 1.92, df = 4, p = 0.13).
